# Distinct and overlapping roles of MutLγ, Mus81-Mms4, and STR in meiotic Holliday junction processing

**DOI:** 10.1038/s41467-026-73888-2

**Published:** 2026-06-02

**Authors:** Lucija Orlić, Adrian Henggeler, Jázmin Nagy, Joao Matos

**Affiliations:** 1 Max Perutz Labs, Vienna BioCenter, Vienna, Austria; 2 University of Vienna, Vienna, Austria; 3 Vienna BioCenter PhD Program, A Doctoral School of the University of Vienna and the Medical University of Vienna, Vienna, Austria

**Keywords:** DNA recombination, Meiosis, Chromosomes

## Abstract

Most meiotic crossovers arise from the nucleolytic resolution of recombination intermediates that ZMM proteins stabilize as double Holliday junctions (dHJs). MutLγ is the nuclease thought to resolve these ZMM-bound dHJs into crossovers, but alternative enzymes - including Mus81-Mms4 and the Sgs1-Top3-Rmi1 (STR) complex - can also process meiotic DNA joint molecules. How ZMM-bound dHJs are preferentially steered toward MutLγ-mediated processing has remained unresolved, in part because experimental systems have been unable to uncouple dHJ resolution from upstream recombination events and downstream cell-cycle progression. To overcome this limitation, we engineered a budding yeast system that stabilizes pre-existing ZMM-bound dHJs, eliminates the continued occurrence of upstream recombination events, and enables conditional pathway-specific resolution without cell-cycle advance. Using this approach, we show that MutLγ is uniquely capable of imposing crossover-specific resolution on ZMM-bound dHJs. In contrast, Mus81-Mms4 and STR can access crossover-designated recombination intermediates but generate mixed or exclusively noncrossover products. We further identify an Sgs1-independent role for Top3-Rmi1 in maintaining ZMM-dHJ architecture and preventing their conversion into aberrant, MutLγ-refractory species. Together, our findings reveal that ZMM proteins establish a hierarchy, rather than absolute selectivity, in dHJ processing, one that favours MutLγ-directed crossovers while preserving alternative resolution routes to safeguard chromosome segregation.

## Abstract (web summary)

Meiotic crossovers ensure accurate chromosome segregation and promote genetic diversity. Here, the authors uncover a key role for Top3-Rmi1 in preserving crossover-precursor architecture and delineate how MutLγ, Mus81-Mms4 and STR differ in processing efficiency and outcome specificity.

## Introduction

Meiotic crossing-over is a fundamental process in which homologous chromosomes reciprocally exchange DNA segments, generating genetic diversity and ensuring accurate segregation during meiosis I^1^. Crossovers arise through homologous recombination, initiated by the programmed formation of DNA double-strand breaks (DSBs)^2^. Following DNA end resection, the resulting 3′ single-stranded tails invade homologous templates to form displacement loops (D-loops), which serve as primers for DNA synthesis^3^. These early recombination intermediates can follow distinct fates: a subset matures into intermediates that commit to the crossover pathway, while the rest of the D-loops are either dismantled into noncrossovers or they mature into intermediates that are processed by structure-selective endonucleases (SSEs), potentially yielding both crossovers and noncrossovers^3^.

The stabilization and maturation of crossover precursors depend on the conserved ZMM (Zip1-4, Msh4-5, Mer3 and Spo16) proteins, a multi-component ensemble that defines the canonical meiotic crossover pathway^4–8^. Collectively, these proteins stabilize D-loops into single-end intermediates (SEIs) and promote the formation of four-armed intermediates containing double Holliday junctions (dHJs)^9,10^. ZMM-bound dHJs are the primary substrates for the MutLγ endonuclease (Mlh1-Mlh3), which is thought to resolve them exclusively into crossovers^11^. Despite extensive genetic evidence linking ZMMs to MutLγ-dependent resolution, the mechanistic basis by which ZMM association directs dHJs toward this specific outcome remains unclear^12,13^. In particular, whether ZMM proteins act by recruiting MutLγ, restricting access to competing pathways, or modulating dHJ topology is not yet understood.

In budding yeast, the Sgs1-Top3-Rmi1 (STR) helicase-topoisomerase complex interfaces with recombination intermediates in ways that underscore the delicate balance of meiotic recombination control^11,14^. STR dismantles extending D-loops, promoting noncrossovers via synthesis-dependent strand annealing and preventing formation of aberrant DNA joint molecules^15–17^. Top3-Rmi1, independently of Sgs1, removes a separate class of aberrant joint molecules in late prophase I^18–20^. Paradoxically, STR is also required for efficient formation of ZMM-bound crossover-designated joint molecules^11,14^. Moreover, it has been proposed to assist MutLγ during late dHJ resolution steps^11,13^, although this remains experimentally untested. Notably, recent findings indicate that ZMM proteins actively protect dHJs from STR-mediated dissolution, suggesting that STR retains the potential to dismantle mature recombination intermediates unless they are stabilized by ZMMs^21–23^. These observations raise a central question: how are the anti- and pro-crossover functions of STR coordinated to preserve, rather than eliminate, crossover-designated intermediates?

While ZMMs and STR interplay with the majority of recombination intermediates, a smaller subset of DNA joint molecules arises independently of these pathways and is primarily processed by the SSE Mus81-Mms4. In mitotically dividing cells - outside the ZMM context - Mus81-Mms4 is thought to cleave dHJs to generate both crossovers and noncrossovers^24,25^. Another SSE, Yen1, acts later in meiosis to resolve persistent recombination intermediates that escape earlier processing^24,26–28^. The activities of these nucleases are tightly controlled by the Polo-like kinase Cdc5, which directly phosphorylates Mus81-Mms4 to stimulate its nuclease activity at the prophase I-to-metaphase I transition^24^ and indirectly regulates Yen1 through Cdc14-dependent dephosphorylation during anaphase^26,27^. Cdc5 activation itself is triggered by the transcription factor Ndt80, marking the transition from meiotic prophase I to metaphase I and coordinating the timely resolution of dHJs and other recombination intermediates^29–31^. While a direct target for Cdc5 phosphorylation in the MutLγ activation pathway remains unidentified, MutLγ activation was shown to depend on a noncanonical interaction between Exo1 and Cdc5, placing MutLγ and Mus81-Mms4 under shared temporal control^32^. This raises the intriguing question of how cells differentiate between these concurrently activated pathways to ensure that distinct subsets of recombination intermediates are processed by the appropriate machinery.

To address how different enzymes engage ZMM-bound dHJs, we developed a system to isolate these intermediates from the flux of ongoing recombination. By combining *ndt80*∆ prophase I arrest with conditional inhibition of DSB formation, we stabilized pools of pre-existing ZMM-bound dHJs. Conditional expression of Cdc5^31^ in specific mutant backgrounds or expression of constitutively active Yen1 (Yen1^ON^)^26^ allowed us to probe the capacities of MutLγ, SSEs, and STR to act on the same defined substrates. Using this approach, we demonstrate that MutLγ has the unique ability to enforce crossover-biased resolution of ZMM-bound dHJs, whereas Mus81-Mms4 and Yen1 act in an unbiased manner, and STR promotes dissolution into noncrossovers. Notably, we find no evidence that STR directly assists MutLγ during resolution. However, our analyses revealed an Sgs1-independent role for Top3-Rmi1 in maintaining dHJ architecture and preventing their conversion into aberrant, MutLγ-refractory species.

Together, our findings define how distinct yet overlapping enzymatic activities act on ZMM-stabilized recombination intermediates, establishing a regulatory hierarchy that favours MutLγ-directed crossovers while preserving alternative routes for genome stability. Given the evolutionary conservation of ZMMs, MutLγ, SSEs and STR, this hierarchical control likely represents a widespread mechanism by which eukaryotic organisms balance crossover assurance with recombination flexibility, ensuring faithful chromosome segregation and fertility across species.

## Results

### Developing a system for the precise analysis of meiotic dHJ processing products

To dissect how different processing pathways act on ZMM-bound recombination intermediates containing dHJs in vivo, we refined the previously described *ndt80*∆ pachytene-arrest/Cdc5-induction system^21,24,31^. In *ndt80*∆ cells, meiotic progression arrests in pachytene with unprocessed joint molecules while DSBs - albeit at lower levels - continue to form^30^, generating a constant flux of early recombination intermediates^21^. We therefore combined *ndt80*∆ with rapamycin-inducible anchor-away depletion of Rec104 (*REC104*^*FRB*^), an essential component of the DSB-forming machinery^33^. In our experimental conditions, most *ndt80*∆ cells arrest meiotic progression at pachytene 6-7 h after transfer to sporulation medium (SPM)^21^. We confirmed that this is also the case for *ndt80∆ REC104*^*FRB*^ strains by FACS analysis of DNA content, to monitor S-phase progression, and by Zip1 staining of chromosome spreads, to assess synapsis (Supplementary Fig. 1a–c). On average, 78% of cells exhibited fully synapsed chromosomes at 6 h, increasing to 85% at 7 h (Supplementary Fig. 1b, c). Accordingly, DSB inhibition was induced at 7 h, when the vast majority of cells had reached pachytene.

**Fig. 1:**
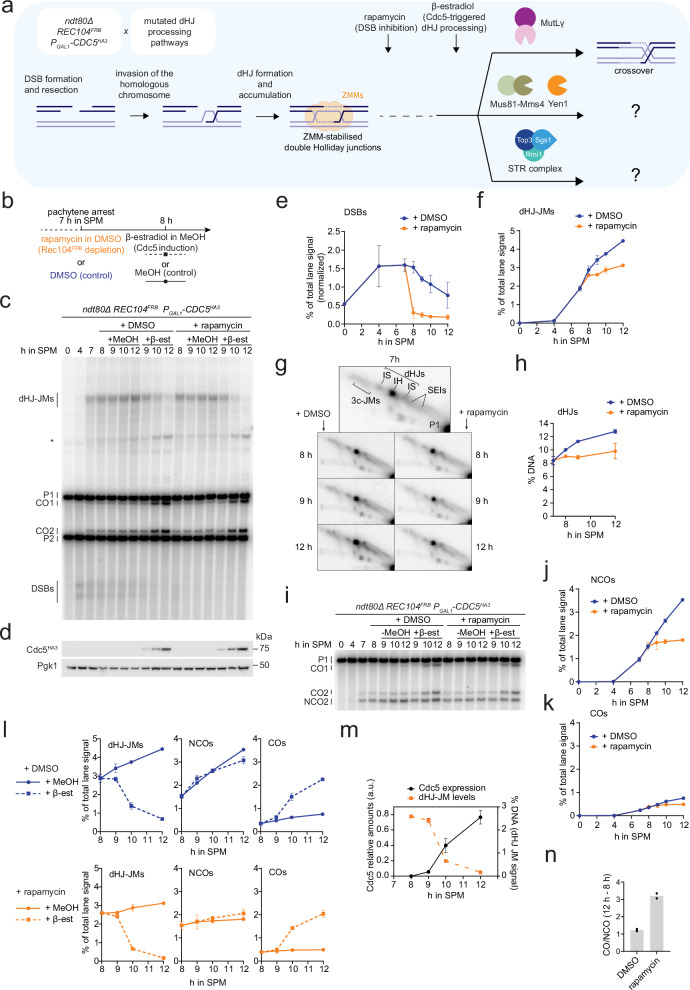
A system to isolate meiotic dHJ-JMs and quantify pathway-specific resolution outcomes. **a** Experimental strategy. To test whether Mus81-Mms4, Yen1^ON^ and Sgs1-Top3-Rmi1 (STR) can process ZMM-bound double Holliday junctions (dHJs), we engineered a system that accumulates meiotic dHJs and enables timed, conditional resolution. *ndt80∆* cells arrest in pachytene with unresolved dHJ joint molecules (dHJ-JMs). After arrest, DSB formation is acutely shut-down by rapamycin-induced depletion of Rec104^FRB^, preventing continued generation of early intermediates. Processing is then triggered by inducible Cdc5 expression (β-estradiol). **b** Experimental timeline. *ndt80∆ REC104*^*FRB*^ *P*_*GAL*_*-CDC5*^*HA3*^ cultures were transferred to sporulation medium (SPM). At 7 h (pachytene arrest), cultures were split and treated with rapamycin (1 μg/mL) or DMSO. At 8 h, each condition was split and treated with β-estradiol (4 μM) or methanol (MeOH). Two biological replicates are performed. **c** Representative physical analysis of recombination at *HIS4::LEU2* recombination hotspot from cells in (b). dHJ-JMs (dHJ-sized joint molecules), asterisk (ectopic COs), P1/P2 (parentals), CO1/CO2 (reciprocal recombinants), DSBs (double-strand breaks). **d** Representative western blot analysis of Cdc5 expression from cells in (**b**). **e** DSBs were quantified from (**c**) and a biological replicate. Data are the mean and range of the percentage of total lane signal (background subtracted). **f** dHJ-JMs were quantified from (**c**) and a biological replicate. Data are the mean and range of the percentage of total lane signal. **g** Representative two-dimensional gel analysis of recombination intermediates at *HIS4::LEU2* from cells in (**b**). P1 (parental), SEIs (single-end intermediates), dHJs (IH, inter-homologue; IS, inter-sister), 3c-JMs (three-chromatid joint molecules). **h** Quantification of dHJs, as described in Supplementary Fig. 1e, from (**g**) and a biological replicate. Data are the mean and range of the percentage of total DNA signal. **i** Representative CO and NCO analysis at *HIS4::LEU2* from cells in (**b**). P1 (parental), CO1/CO2 (reciprocal recombinants), NCO2 (noncrossover recombinant). Quantification of NCOs (**j**) and COs (**k**) from (**i**) and a biological replicate, as in (**f**). **l** dHJ-JMs, NCOs and COs were quantified as in (**f**), (**j**), (**k**). **m** Cdc5 levels and dHJ-JMs were quantified from +rapamycin +β-estradiol samples from (**c**). Data are the mean and range of two independent experiments. **n** CO:NCO ratio generated upon Cdc5 induction under DSB-shutdown conditions. CO and NCO production was calculated as the increase from 8 h to 12 h (∆12h-8h) using data in (**l**); the ratio was computed as ∆CO/∆NCO. Source data are provided as a Source data file.

After arresting cells in pachytene and inhibiting DSB formation, we induced Cdc5 expression with β-estradiol to trigger dHJ processing (experimental scheme in Fig. 1a, b). Meiotic DNA replication was monitored in all experiments as a proxy for synchronous entry into meiosis and to ensure comparable S-phase kinetics across cultures and experiments. Time courses deviating from the expected replication profile were excluded from further analyses. Recombination dynamics at the *HIS4::LEU2* recombination hotspot were monitored by Southern-blotting-based physical assays, as previously described^10,21,34^.

In wild-type cultures, DSBs peak ~3–4 h after transfer to SPM, followed by a peak in joint molecules ~1 h later^35,36^. Joint molecules are subsequently processed into crossovers, such that recombination is completed by ~8 h in SPM^35,36^. In contrast, in our system DSB levels remained elevated at 7 h and declined only gradually over time (control DMSO-treated cultures; Fig. 1c–e, blue line). Notably, Rec104 anchor-away led to a rapid reduction in DSB signal to near background levels within ~1 h (Fig. 1e, orange line; Supplementary Fig. 1d). Consistent with this, dHJ-containing joint molecules (dHJ-JMs) continued to accumulate in DMSO-treated cultures, whereas after Rec104 anchor-away dHJ-JM levels plateaued (Fig. 1c, f). Two-dimensional gel electrophoresis confirmed that the predominant species under both conditions were four-armed intermediates containing dHJs, with inter-homologue dHJ-JMs comprising the majority (Fig. 1g, h; Supplementary Fig. 1e, f). The reduced accumulation of dHJ-JMs after DSB inhibition indicates that late forming DSBs can still be steered towards the ZMM pathway.

Importantly, analysis of recombination outcome (crossover vs non-crossover) showed that DSB inhibition also strongly reduced the continued formation of noncrossovers (NCOs), which plateaued shortly after rapamycin addition (Fig. 1i, j). These data indicate that the continued rise in NCOs in control DMSO-treated *ndt80*∆ cultures primarily reflects processing of newly formed DSBs rather than processing of the stable dHJ-JM pool. Ndt80-independent crossovers (COs), which form at low levels at *HIS4::LEU2*^30^, similarly showed little additional accumulation after DSB shutdown (Fig. 1i, k), consistent with upstream recombination being paused while a defined cohort of stable intermediates persisted.

Having established that acute DSB shutdown in pachytene-arrested cultures selectively stabilises dHJ-JMs, we next examined how Cdc5 induction impacts their processing and recombination outcome. Upon Cdc5 expression (Fig. 1d, l), analogous to the rapid wild-type JM-to-CO conversion^35^, dHJ-JM signals declined sharply to near-background levels, most prominently in rapamycin-treated cultures in which new DSB formation was suppressed (Fig. 1c, l, m; Supplementary Fig. 1e–g). Notably, dHJ-JM loss was accompanied by a marked and specific rise in CO formation (Fig. 1i, l).

Because dHJ-JM abundance can vary between experiments and genetic backgrounds, we quantified recombination outcomes using a CO:NCO ratio. CO and NCO values were defined as the increase between 8 h in SPM (time of Cdc5 induction) and 12 h, thereby capturing products formed after induction of Cdc5 expression. This approach largely excludes contributions from the processing of nascent intermediates, such as synthesis-dependent strand annealing, and focuses on COs and NCOs derived from late joint molecule resolution. Under DSB-inhibited conditions, this metric provides a robust readout of CO-biased resolution (CO:NCO > 1) and enables direct comparisons across strains and perturbations (Fig. 1n). Together, these results confirm that CO-biased resolution is preserved when new DSB formation is shut off, establishing this approach as a platform to compare how defined pools of meiotic dHJ-JMs are processed by alternative pathways.

### MutLγ, but not SSEs, drives crossover-biased resolution

**Fig. 2:**
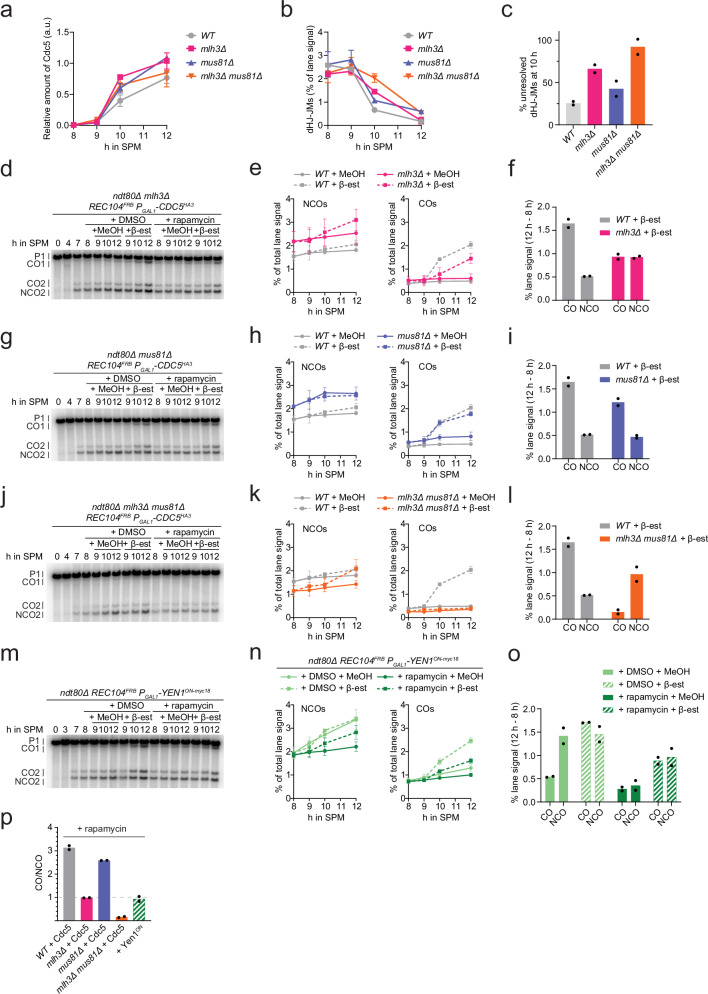
Mus81-Mms4 and Yen1^ON^ can process crossover-designated dHJ-JMs, but only MutLγ imposes crossover-biased resolution. **a** *REC104*^*FRB*^ *P*_*GAL*_*-CDC5*^*HA3*^ *ndt80∆* cells carrying *mlh3∆*, *mus81∆*, or *mlh3∆ mus81∆* were processed as in Fig. 1b, *n* = 2 per genotype. Relative Cdc5 abundance in +rapamycin +β-estradiol samples was quantified from western blots in which all samples were loaded on the same gel. Data is plotted as mean and range. **b** dHJ-JMs quantification at *HIS4::LEU2* from +rapamycin +β-estradiol samples in (**a**) analysed by Southern blotting (Fig. 1c; Supplementary Fig. 2a, e, i and biological replicates), and plotted as % total lane signal (mean and range). **c** dHJ-JMs remaining 2 h after Cdc5 induction (10 h in SPM) calculated from (**b**), plotted as mean percentage of the dHJ-JM signal at 8 h (time of β-estradiol addition). **d** Representative CO/NCO analysis at *HIS4::LEU2* from *mlh3∆* cells in (**a**). P1 (parental), CO1/CO2 (reciprocal recombinants), NCO2 (noncrossover recombinant). **e** Quantification of NCOs and COs from *mlh3∆* under DSB-shutdown ( + rapamycin; magenta) from (**d**) compared with *WT* (+rapamycin; grey; from Fig. 1l), plotted as % total lane signal (mean and range). **f** CO and NCO production after DSB shutdown and Cdc5 induction in *mlh3∆*, calculated from (**e**) and plotted as mean ∆(12 h − 8 h). **g** Representative CO/NCO analysis at *HIS4::LEU2* from *mus81∆* cells in (**a**). **h** Quantification of NCOs and COs from *mus81∆* under DSB-shutdown ( + rapamycin; blue) from (**g**), as in (**e**). **i**, CO and NCO production in *mus81∆*, from (**h**), as in (**f**). **j** Representative CO/NCO analysis at *HIS4::LEU2* from *mlh3∆ mus81∆* cells in (**a**). **k** Quantification of NCOs and COs from *mlh3∆ mus81∆* under DSB-shutdown ( + rapamycin; orange) from (**j**), as in (**e**). **l** CO and NCO production in *mlh3∆ mus81∆*, from (**k**), as in (**f**). **m** Representative CO/NCO analysis at *HIS4::LEU2* from experiment as in Fig. 1^21^ with *P*_*GAL1*_*-YEN1*^*ON-myc18*^ instead of *P*_*GAL1*_*-CDC5*^*HA3*^, *n* = 2. **n** Quantification of NCOs and COs from (**m**) and a biological replicate, plotted as % total lane signal (mean and range). **o** CO and NCO production after Yen1^ON^ induction, calculated as ∆(12 h − 8 h) from (**n**), as in (**f**). **p** CO:NCO ratios for products formed under DSB shutdown and β-estradiol addition (8–12 h in SPM), calculated using ∆CO/∆NCO values from (**f**), (**i**), (**l**) and (**o**), plotted as means (*n* = 2). Values > 1 indicate CO bias; ~1 indicates unbiased outcomes; <1 indicates NCO bias. Source data are provided as a Source data file.

We next asked which joint molecule-processing pathways can engage meiotic dHJ-JMs and how they shape recombination outcome. We focused first on the two principal meiotic resolvases, MutLγ and Mus81-Mms4, analysing strains lacking *MLH3*, *MUS81*, or both. To ensure that differences in dHJ-JM turnover reflected altered processing rather than variation in the trigger, we monitored Cdc5 accumulation and confirmed that Cdc5 expression was at least as high as in the wild-type for all mutant backgrounds (Fig. 2a).

Following Cdc5 induction, dHJ-JMs declined in all strains, including *mlh3*∆ *mus81*∆ double mutants, although with distinct kinetics (Fig. 2b; Supplementary Fig. 2a–l). 2 h after Cdc5 induction, wild-type cultures retained ~25% of dHJ-JMs (Fig. 1c), whereas *mus81*∆ retained ~40% and *mlh3*∆ ~70%; in *mlh3*∆ *mus81*∆, ~90% persisted (Fig. 2c). This ordering is consistent with MutLγ providing the dominant Cdc5-responsive activity, with Mus81-Mms4 contributing a partially redundant pathway that becomes more evident when MutLγ is absent. The delayed decline of dHJ-JMs in the double mutant at later time points (Fig. 2b) further suggests that additional pathways can access these intermediates when both resolvases are absent.

We next examined recombination outcomes after Cdc5 expression (8–12 h in SPM). In *mlh3*∆, CO and NCO products accumulated to similar levels, consistent with a loss of crossover-biased resolution (Fig. 2d–f; Supplementary Fig. 2a–d). By contrast, *mus81*∆ cultures retained crossover-biased outcomes, although total CO levels were reduced (Fig. 2g–i; Supplementary Fig. 2e–h). In contrast, *mlh3*∆ *mus81*∆ double mutants failed to generate detectable COs, with products instead appearing as NCOs (Fig. 2j–l; Supplementary Fig. 2i-l). This NCO formation suggested engagement of an additional late-acting pathway, raising the possibility that STR-mediated dissolution contributes when both nucleases are absent.

To complement the *mlh3*∆ loss-of-function analysis, which implies that SSE-dependent processing in the absence of MutLγ does not strongly favour COs, we next asked whether forced SSE activation can impose CO bias on crossover-designated substrates even when MutLγ is intact. We therefore turned to Yen1^ON^ ^26^, a constitutively active Yen1 variant that we previously used to drive JM turnover and that has been reported to elevate COs without a corresponding increase in NCOs^28^. This phenotype has been interpreted as evidence that late meiotic intermediates are intrinsically pre-patterned to yield predominantly CO products upon cleavage. However, in standard *ndt80*∆ arrested cells, ongoing DSB formation sustains the appearance and turnover of nascent intermediates, complicating attribution of CO/NCO output to the stable, ZMM-bound dHJ-JM pool.

We therefore expressed Yen1^ON^ within our DSB-shutdown framework, restricting its activity to a defined cohort of pre-existing, ZMM-bound dHJ-JMs. Under these DSB-depleted conditions, Yen1^ON^ generated a mixture of COs and NCOs (Fig. 2m–o), in contrast to the CO skew observed in control-treated cultures where new intermediates continue to arise^21,28^. Thus, when assayed on an isolated pool of crossover-designated dHJ-JMs, Yen1^ON^ shows little intrinsic CO bias, arguing against a general pre-patterning model in which late intermediates obligatorily yield CO products upon endonucleolytic cleavage. Consistent with this, CO:NCO ratios separated pathway outputs cleanly: CO-biased outcomes (CO:NCO > 1) were observed only when MutLγ was present, whereas Mus81-Mms4 (inferred from *mlh3*∆) and Yen1^ON^ produced approximately unbiased products (CO:NCO ~1) (Fig. 2p). Together, these data indicate that multiple activities can process ZMM-bound meiotic dHJ-JMs, but MutLγ uniquely imposes CO-biased resolution, an insight that becomes apparent when late JM processing is examined independently of ongoing recombination.

### STR functions as a fail-safe in the processing of crossover-designated dHJ-JMs

The delayed yet detectable formation of NCO recombinants upon Cdc5 induction in *mlh3*∆ *mus81*∆ cells suggested that an additional late-acting pathway can process dHJ-JMs when both principal resolvases are absent. A prime candidate is the STR complex, which is essential early in recombination for establishing crossover-designated intermediates^14,15,17–19,37,38^, yet also retains the potential to dismantle dHJ-JMs upon acute perturbation of ZMM function^21,22^. To test whether STR accounts for the residual processing observed in *mlh3*∆ *mus81*∆, we implemented conditional, late meiotic depletion of individual STR subunits.

**Fig. 3:**
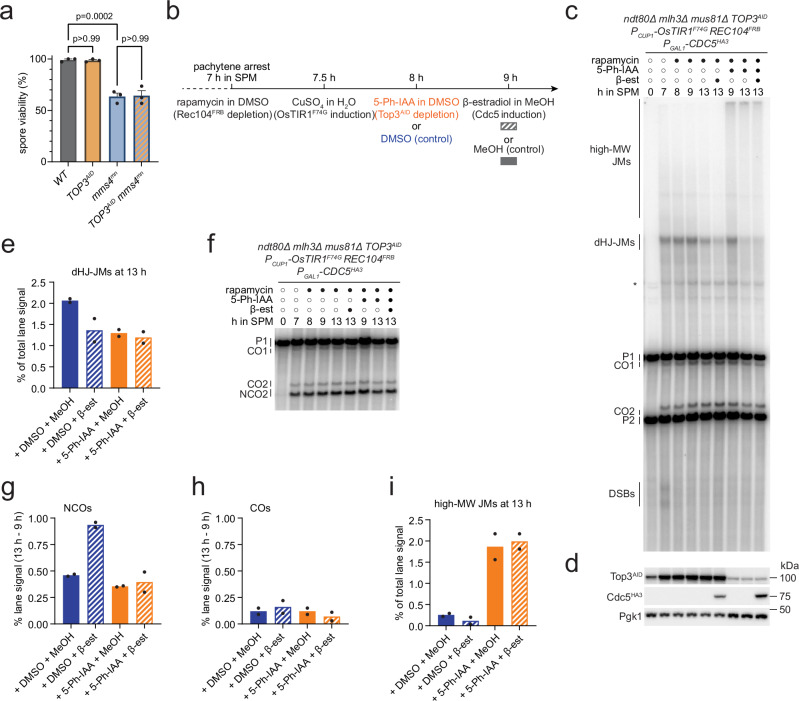
Top3 is required for STR-dependent dissolution of crossover-designated joint molecules into noncrossovers when nucleolytic resolution is disabled. **a** Spore viability of strains with the indicated genotypes. Sporulated cultures were dissected 48 h after meiosis induction on SPM plates (30 °C). A total of 216 spores per strain were scored across three independent experiments. Data are mean ± SEM (one-way ANOVA, *p* < 0.0001; Tukey’s multiple-comparisons test). mn (meiotic null). **b** Experimental scheme for combining late Top3^AID^ depletion with DSB shutdown and Cdc5 induction. *ndt80∆ REC104*^*FRB*^ *P*_*GAL*_*-CDC5*^*HA3*^ *mlh3*∆ *mus81*∆ *TOP3*^*AID*^ cells expressing P_*CUP1*_-*OsTIR1*^*F74G*^ were transferred to SPM. Rapamycin (1 μg/mL) was added at 7 h to inhibit DSB formation, followed by CuSO_4_ (50 μM) at 7.5 h to induce OsTIR1^F74G^. At 8 h, cultures were split and treated with 5-Ph-IAA (75 μM) to trigger Top3^AID^ depletion or with DMSO (control). At 9 h, cultures were split again and treated with β-estradiol (4 μM) to induce Cdc5 or with methanol (MeOH) as control. Two biological replicates are performed. **c** Representative physical analysis of recombination intermediates at *HIS4::LEU2* for the experiment in (**b**). dHJ-JMs (dHJ-sized joint molecules), high-MW JMs (high-molecular-weight joint molecules), asterisk (ectopic COs), P1/P2 (parentals), CO1/CO2 (reciprocal recombinants), DSBs (double-strand breaks). **d** Representative western blot analysis of Top3^AID^ depletion and Cdc5 expression from cells in (**b**). **e** Quantification of dHJ-sized JMs at 13 h in SPM from (**c**) and a biological replicate, plotted as the mean percentage of total lane signal. **f**, Representative CO/NCO analysis at *HIS4::LEU2* from cells in (**b**). P1 (parental), CO1/CO2 (reciprocal recombinants), NCO2 (noncrossover recombinant). **g** NCO formation after Cdc5 induction, quantified and calculated from (**f**) and plotted as mean ∆(13 h − 9 h). **h** CO formation after Cdc5 induction, as in (**g**). **i** Quantification of high-MW JMs at 13 h in SPM from (**c**), as in (**e**). Source data are provided as a Source data file.

To preserve STR function during joint molecule formation while enabling acute removal at later stages, we tagged Sgs1, Top3, and Rmi1 with auxin-inducible degrons (AID-myc9; hereafter AID) and validated that the tags did not grossly compromise meiotic function. Spore viability assays served as a sensitive proxy for functional integrity, particularly because loss of STR becomes strongly deleterious when Mus81-Mms4 activity is simultaneously impaired^37,38^. Accordingly, we tested the AID-tagged alleles both alone and in *mus81*∆ or *mms4*^*mn*^ backgrounds (mn; meiotic null). Consistent with partial impairment, *SGS1*^*AID*^ *mus81*∆ showed slightly reduced spore viability compared to *mus81*∆ alone (Supplementary Fig. 3a), whereas *TOP3*^*AID*^ and *RMI1*^*AID*^ had no detectable impact on spore viability, even in combination with *mms4*^*mn*^ (Fig. 3a; Supplementary Fig. 3b). We also observed that *ndt80*∆ *REC104*^*FRB*^ *OsTIR1*^*F74G*^ strains carrying the AID-tagged alleles reached full synapsis with comparable efficiency to untagged controls (Supplementary Fig. 3c; Supplementary Fig. 1c). In light of the spore viability data, we focused subsequent mechanistic analyses primarily on Top3.

Next, we combined *TOP3*^*AID*^ with *mlh3*∆ *mus81*∆ (Supplementary Fig. 3c) and applied auxin-mediated degradation after efficient DSB shutdown, thereby allowing normal establishment of meiotic recombination intermediates before perturbing STR function (Fig. 3b, c; Supplementary Fig. 3d, e). Top3^AID^ levels decreased markedly within ~1 h of 5-Ph-IAA addition, although a residual fraction remained detectable throughout the experiment (Fig. 3d, Supplementary Fig. 3e). We then induced Cdc5 to trigger dHJ-JM processing. As hypothesised, in Top3^AID^-depleted cultures, Cdc5 induction failed to drive turnover of dHJ-JMs relative to MeOH-treated controls (Fig. 3c, e; orange columns). Correspondingly, neither NCOs nor COs increased (Fig. 3f–h; orange columns). Conversely, in the presence of Top3^AID^ dHJ-JMs were converted into NCOs (Fig. 3c, e–h; blue columns). These results indicate that the NCOs that still arise in the *mlh3*∆ *mus81*∆ background depend on Top3, implicating STR as the pathway responsible for the residual processing of crossover-designated intermediates when MutLγ and Mus81-Mms4 are absent.

Unexpectedly, we noticed that Top3^AID^ depletion caused a pronounced redistribution of joint molecule species. dHJ-JM signals were reduced (Fig. 3c, e), coincident with the emergence of a high-molecular-weight smear (high-MW JMs) near the top of the blot (Fig. 3c, i), consistent with accumulation of aberrant joint molecules. This pattern suggests that, beyond promoting noncrossover formation in the *mlh3*∆ *mus81*∆ context, Top3 also plays an additional role in maintaining the architecture/integrity of crossover-designated intermediates. This observation is in agreement with previous work implicating Top3 in suppressing meiosis I catastrophe in *ndt80*∆-arrest-release experiments^18^.

### Top3-Rmi1 preserves the architecture of crossover-designated dHJ-JMs

**Fig. 4:**
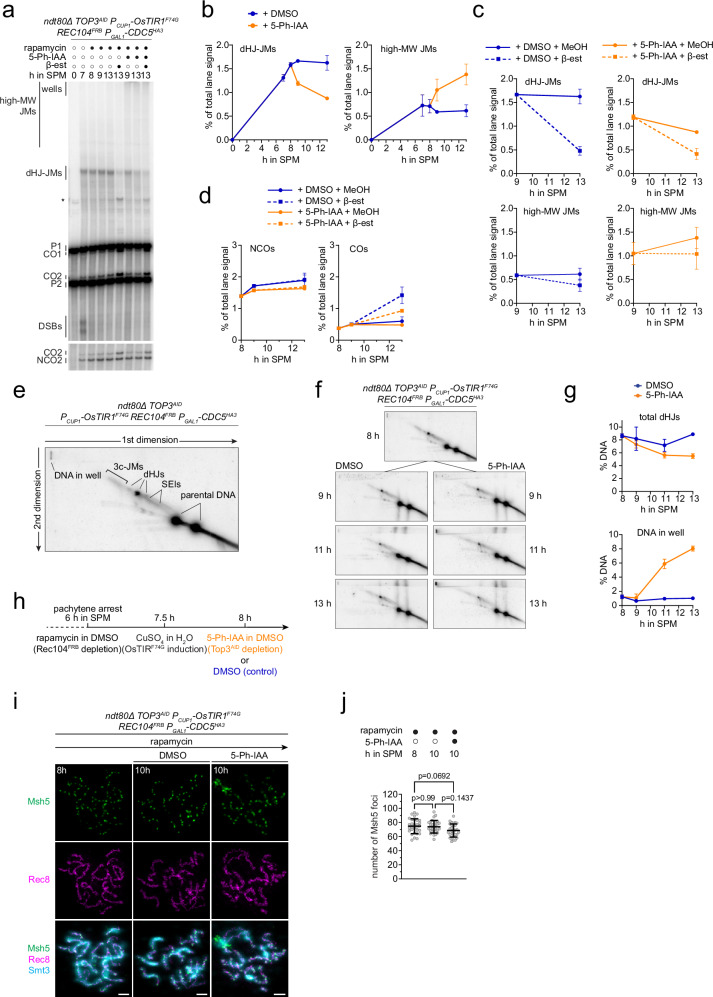
Top3 preserves dHJ-JMs during pachytene to sustain efficient MutLγ-dependent crossover formation. **a** Representative physical analysis of recombination at *HIS4::LEU2* in *TOP3*^*AID*^ cells, processed as in Fig. 3b. Top, XhoI digest for joint molecule analysis; bottom, XhoI + NgoMIV digest for CO/NCO analysis. dHJ-JMs (dHJ-sized joint molecules), high-MW JMs (high-molecular-weight joint molecules), asterisk (ectopic COs), P1/P2 (parentals), CO1/CO2 (reciprocal recombinants), DSBs (double-strand breaks), NCO2 (noncrossover recombinant). Representative of two independent experiments (*n* = 2). **b** dHJ-sized JMs (left) and high-MW JMs (right) were quantified from MeOH-treated samples from (**a**) and a biological replicate and plotted as % total lane signal (mean and range). **c** Quantification of dHJ-sized and high-MW JMs after β-estradiol–induction (9 h in SPM onwards) from (**a**) and a biological replicate, as in (**b**). **d** Quantification of COs and NCOs after 5-Ph-IAA addition from CO/NCO blots in (a, bottom) and a biological replicate, plotted as % total lane signal (mean and range). **e** Representative two-dimensional (2D) gel recombination analysis. As in Fig. 1g but including the well/gel origin to visualize accumulation of low-mobility, aberrant intermediates. **f** 2D analysis of recombination intermediates at *HIS4::LEU2* in *TOP3*^*AID*^ cells from (**a**). Only MeOH-treated samples (no Cdc5 induction) were analysed. **g** Quantification from (**f**) and a biological replicate, plotted as % total DNA (mean and range). Top, canonical dHJ signal; bottom, DNA retained at the well/gel origin. **h** Experimental scheme for meiotic chromosome spreads. Cells were transferred to SPM to induce meiosis; rapamycin was added at 6 h to inhibit DSB formation. CuSO_4_ (50 μM) was added at 7.5 h to induce OsTIR1^F74G^, and cultures were split at 8 h for 5-Ph-IAA (75 μM)–mediated depletion or DMSO control. **i** Representative STED images of chromosome spreads from (**h**), immunostained for Msh5 (green), Rec8 (magenta) and Smt3 (cyan). Scale bars, 1 μm. **j** Quantification of Msh5 foci from (**i**). Data are mean ± SD; *n* = 30 nuclei per condition. Kruskal–Wallis test (*p* = 0.0471) with Dunn’s multiple-comparisons test. Source data are provided as a Source data file.

To determine if the Top3-dependent changes in joint molecule composition impact resolution when the canonical resolvases are intact, we depleted Top3^AID^ in an otherwise wild-type background for MutLγ and Mus81-Mms4. As in *mlh3*∆ *mus81*∆, acute Top3^AID^ depletion reduced the signal of dHJ-sized joint molecules and was accompanied by the accumulation of high-MW species (Fig. 4a, b; Supplementary Fig. 4a–c). Upon subsequent Cdc5 induction, both dHJ-sized and high-MW JM signals declined (Fig. 4c), and recombination products were dominated by COs (Fig. 4d). However, CO levels were reduced relative to non-depleted controls (Fig. 4a, bottom; Fig. 4d), suggesting that Top3 contributes to maintaining a productive pool of crossover-designated intermediates rather than being required for CO bias per se.

We next asked whether this phenotype reflects loss of STR function as a complex, or a specific requirement for Top3. Depletion of Rmi1^AID^ closely phenocopied Top3^AID^ depletion: dHJ-sized JMs were reduced and high-MW JMs accumulated prominently, yet Cdc5 induction still produced CO-biased outcomes (Supplementary Fig. 4d–j). In contrast, depletion of Sgs1^AID^ did not reproduce the phenotype of *TOP3*^*AID*^/*RMI1*^*AID*^ (Supplementary Fig. 5a–c). Potentially because the AID tag partially compromises Sgs1 function (Supplementary Fig. 3a), dHJ-sized JMs appeared unstable throughout the pachytene arrest in *SGS1*^*AID*^ strains, even in the absence of auxin-mediated depletion (Supplementary Fig. 5a–c). Notably, acute Sgs1^AID^ depletion did not further reduce dHJ-JM levels, nor did it trigger the characteristic high-MW JM accumulation seen after Top3^AID^ or Rmi1^AID^ depletion (compare Supplementary Fig. 5c to Fig. 4b, Supplementary Fig. 4h). Moreover, the high-MW signal in *SGS1*^*AID*^ samples was broader in size distribution, whereas Top3^AID^/Rmi1^AID^ depletion produced a more concentrated accumulation immediately below the wells (Fig. 4a; Supplementary Fig. 4d; Supplementary Fig. 5a; proportion of JM signal at wells quantified in Supplementary Fig. 5d). Despite partial impairment of Sgs1 function in *SGS1*^*AID*^ cells, MutLγ-dependent crossovers were still formed, and pachytene-specific depletion of Sgs1^AID^ did not measurably alter CO/NCO outcomes (Supplementary Fig. 5e). Together, these data are consistent with a Top3-Rmi1-dependent, Sgs1-independent role in preserving the composition/architecture of crossover-designated recombination intermediates. We note, however, that the hypomorphic behaviour of the *SGS1*^*AID*^ allele limits the strength of this inference, precluding definitive conclusions about Sgs1.

To verify that the decrease in the dHJ-sized joint molecule signal upon Top3^AID^ depletion reflects loss of bona fide dHJ-JMs, we analysed recombination intermediates by two-dimensional gel electrophoresis. This confirmed a reduction in canonical dHJ-JM species and revealed a substantial fraction of DNA retained near the well of the gel (up to ~8%), consistent with accumulation of highly branched or otherwise abnormal structures (Fig. 4e–g).

Finally, we asked whether Top3^AID^ depletion measurably affects the stable association of ZMM proteins with crossover-designated intermediates and/or chromosome synapsis. Immunostaining of chromosome spreads for the ZMM protein Msh5 and the axis marker Rec8 revealed only a modest, non-statistically significant reduction in Msh5 foci upon Top3^AID^ depletion (Fig. 4h–j; Supplementary Fig. 5f). Likewise, staining for Zip1, did not reveal obvious defects in synapsis (Supplementary Fig. 5g–h). Strikingly, however, staining for Smt3/SUMO – which is known to decorate the synaptonemal complex^39^—frequently changed in Top3^AID^-depleted nuclei: prominent punctate speckles appeared superimposed on the characteristic linear Smt3 signal along synapsed chromosomes (Supplementary Fig. 5g, h). Thus, Top3 is largely dispensable for synapsis and for stable ZMM association with meiotic chromosomes, but it may directly or indirectly influence SUMO accumulation and/or the distribution of SUMOylated proteins within the synaptonemal complex.

Together, these observations support a model in which Top3-Rmi1 acts during late prophase I to preserve the architecture of a subset of crossover-designated dHJ-JMs, limiting their diversion into aberrant joint molecules that reduce the pool of substrates available for efficient MutLγ-mediated crossover formation. Notably, the reduced MutLγ-dependent processing of a subset of intermediates appears uncoupled from ZMM binding, consistent with Top3-Rmi1 acting primarily on dHJ structure or topology rather than on ZMM recruitment.

### Top3 restrains aberrant remodelling of crossover-designated intermediates

**Fig. 5:**
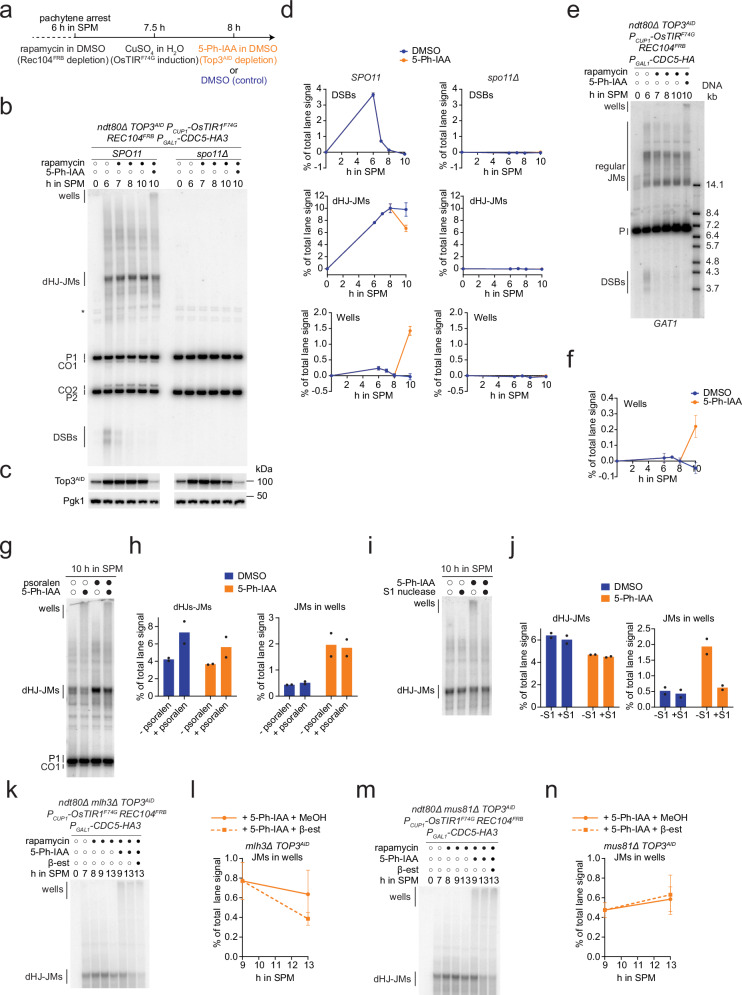
Top3 maintains the architecture of a subset of crossover-designated dHJ-JMs during pachytene. **a** Experimental scheme to extend the DSB-shutdown interval prior to Top3^AID^ depletion. Cells were transferred to SPM to induce meiosis; rapamycin was added at 6 h to inhibit DSB formation. CuSO_4_ (50 μM) was added at 7.5 h to induce OsTIR1^F74G^, and cultures were split at 8 h for 5-Ph-IAA (75 μM)–mediated Top3^AID^ depletion or DMSO control, yielding a 2 h window of DSB inhibition before Top3^AID^ depletion. Two biological replicates are performed. **b** Representative physical analysis of recombination at *HIS4::LEU2* from the experiment in (**a**), for the indicated genotypes. Genomic DNA was prepared in agarose plugs. **c** Representative western blot analysis of Top3^AID^ depletion from experiment in (**a**, **b**). **d** Quantification of DSBs, dHJ-sized JMs (dHJ-JMs), and high-molecular-weight material retained in the wells (Wells) from (**b**) and a biological replicate, plotted as % total lane signal (mean and range). Southern blot (**e**) and quantification (**f**) of DNA JMs in wells at the native *GAT1* hotspot in *SPO11* cells from (**a**), prepared as in (**b**), plotted as in (**d**). P (parental DNA), DSBs (double-strand breaks). Southern blot (**g**) and quantification (**h**) of joint molecule species showing the effect of psoralen crosslinking on detection of joint molecule species. Cells from an experiment as in (**a**) were collected at 10 h in SPM with or without psoralen crosslinking, then processed as in (**b**). Data is the mean of two independent experiments. **i** S1 nuclease sensitivity of joint molecules. Psoralen-crosslinked samples from (**g**) were treated with S1 nuclease or mock treated, followed by analysis at *HIS4::LEU2*. **j** Quantification of joint molecule species from (**i**), plotted as in (**h**). **k** Representative Southern blot of joint molecules at *HIS4::LEU2* in *mlh3∆ TOP3*^*AID*^ cells processed as in Fig. 3b (*n* = 2). **l** Quantification of DNA JMs in wells from (**k**) and Supplementary Fig. 7a, plotted as in (**d**). Solid lines, MeOH control; dotted lines, β-estradiol-induced Cdc5. **m** As in (**k**), for *mus81∆ TOP3*^*AID*^ cells. **n** Quantification of DNA JMs in wells from (**m**) and Supplementary Fig. 7e, plotted as in (**d**). Source data are provided as a Source data file.

Top3^AID^ depletion consistently triggered the appearance of high-MW joint molecules, raising the question of how these species arise. We considered three non-mutually exclusive possibilities: (i) incomplete DSB shutdown, such that residual breaks engage in aberrant recombination; (ii) recombination-independent DNA entanglements; or (iii) aberrant remodelling of pre-existing dHJ-JMs, for example through collision with other DNA-based processes such as transcription. To distinguish between these models, we (a) extended the interval between DSB shutdown and Top3^AID^ depletion to maximize suppression of new break formation (Fig. 5a), and (b) tested Top3^AID^ depletion in a *spo11∆* background, which abolishes meiotic DSB formation and thus recombination.

To maximise recovery of potentially fragile high-MW species, we prepared genomic DNA in agarose plugs and monitored joint molecules at the engineered *HIS4::LEU2* hotspot (Fig. 5b–d). No JM species were detected in *spo11∆* cells, with or without Top3^AID^ depletion (Fig. 5b; quantified in Fig. 5d), indicating that high-MW JMs require Spo11-dependent meiotic recombination and do not reflect generic DNA entanglements. Importantly, in *SPO11* cells, high-MW JMs still accumulated upon Top3^AID^ depletion even when the DSB-shutdown interval was prolonged (Fig. 5b, e; quantified in Fig. 5d, f). As observed before (Fig. 4a), the Top3^AID^-dependent high-MW JM signal was concentrated just below the wells, consistent with the emergence of aberrant structures of limited gel mobility.

We further analysed these same plug-derived samples by two-dimensional gel electrophoresis to better resolve recombination intermediates (Supplementary Fig. 6a). Single-end intermediate (SEI) species were detected predominantly prior to rapamycin addition and declined thereafter with similar kinetics irrespective of Top3^AID^ depletion (Supplementary Fig. 6a, b). In contrast, two- and three-chromatid joint molecules (dHJs and 3c-JMs) remained stable in DMSO-treated cultures but decreased upon Top3^AID^ depletion, coincident with the accumulation of high-MW DNA trapped at the gel origin/within the wells (Supplementary Fig. 6a, b). Together, these results suggest that Top3 acts during pachytene to preserve the integrity of a subset of pre-established dHJ-JMs and to prevent their conversion into aberrant species.

We next asked whether this phenotype is restricted to the *HIS4::LEU2* engineered recombination hotspot. Analysing the native *GAT1* locus in *SPO11* cells revealed the same Top3^AID^-dependent accumulation of high-MW material concentrated just below the wells (Fig. 5e, f), indicating that Top3 suppresses aberrant JM formation in a locus-independent manner.

In mitotically dividing cells, many phenotypes of *top3*∆ mutants are attributed to toxic recombination intermediates generated by unrestrained Sgs1 activity^40^. We therefore asked whether Sgs1 similarly drives high-MW JM accumulation upon Top3^AID^ depletion in meiosis. However, simultaneous depletion of Sgs1 and Top3 (*SGS1*^*AID*^ *TOP3*^*AID*^) did not suppress the high-MW JMs, which accumulated to comparable levels in *TOP3*^*AID*^ single and *TOP3*^*AID*^ *SGS1*^*AID*^ double mutants (Supplementary Fig. 6c–e). Thus, the formation of aberrant high-MW JMs upon Top3^AID^ loss does not simply reflect Sgs1-dependent toxicity.

### High-MW joint molecules contain ssDNA

To gain insight into the nature of the high-MW JMs that accumulate upon Top3^AID^ depletion, we first asked whether their detection depends on psoralen, which forms covalent interstrand crosslinks that prevent DNA branch migration/dissociation during sample processing^34^ (Fig. 5g). Whereas dHJ-JM signals were enhanced by crosslinking, the high-MW material near the wells was detected with similar efficiency in crosslinked and non-crosslinked samples (Fig. 5g–h), indicating that these species are not preferentially revealed by DNA interstrand stabilization.

We next tested whether high-MW JMs contain single-stranded DNA. Top3^AID^-depleted samples were treated with S1 nuclease, which cleaves DNA with single-strand discontinuities^41^ (Fig. 5i). While canonical dHJ-JMs were largely resistant under these conditions, the high-MW signal in the wells decreased ~3-fold following S1 treatment (Fig. 5i–j), indicating that these aberrant JM species are enriched for ssDNA-containing regions.

### Mus81-Mms4, but not MutLγ, partially processes aberrant high-MW joint molecules

In *TOP3*^*AID*^ and *RMI1*^*AID*^ strains, Cdc5 induction partially reduced the high-MW JM signal (Fig. 4c; Supplementary Fig. 4i), suggesting that at least one Cdc5-responsive nuclease can act on these structures. To determine whether aberrant high-MW JMs are substrates for MutLγ and/or Mus81-Mms4, we repeated the Top3^AID^-depletion experiments in *mlh3*∆ and *mus81*∆ backgrounds, respectively (Fig. 5k–n; Supplementary Fig. 7a–h).

High-MW JMs were partially resolved only when Mus81-Mms4 was present: detectable processing was observed in *mlh3*∆ cells but was lost in *mus81*∆ cells, indicating that MutLγ alone is insufficient to cleave these aberrant species (Fig. 5l, n). In contrast, dHJ-sized JMs could be processed by either pathway, although MutLγ-dependent turnover appeared slightly less efficient under Top3^AID^ depletion (Supplementary Fig. 7c, g). Notably, Top3^AID^ depletion did not alter the characteristic outcome signatures of each resolvase: following Cdc5 induction, Mus81-Mms4 generated mixed CO and NCO products, whereas MutLγ-mediated resolution remained strongly CO-biased (Supplementary Fig. 7d, h).

In summary, the data above indicate that Top3-Rmi1 is required to prevent the conversion of a subset of dHJ-JMs into MutLγ-refractory species. At first glance, the coexistence of two behaviours, one JM fraction becoming high-MW and poorly processed by MutLγ, while another remains competent for MutLγ-dependent CO-biased resolution, could be explained by incomplete depletion of Top3^AID^ or Rmi1^AID^. However, this interpretation is unlikely since depletion of Top3^AID^ fully abolished JM processing and NCO formation in *mlh3*∆ *mus81*∆ mutants (Fig. 3e, g), arguing that functional Top3-Rmi1 is reduced to near-background levels with the AID system. We therefore favour a model in which conditional Top3-Rmi1 loss selectively exposes a subset of recombination intermediates to aberrant remodelling by other cellular activities, diverting them into ssDNA-rich configuations that are refractory to MutLγ yet partially susceptible to SSE processing. Under this view, Top3-Rmi1 acts as an architectural safeguard that maintains resolvase-competent dHJ-JMs in the face of competing DNA transactions, thereby preserving the effective substrate pool available for productive MutLγ-mediated crossover resolution.

## Discussion

A long-standing challenge in dissecting meiotic Holliday junction processing is separating late JM resolution from the upstream flux of DSB formation and nascent intermediates, and from downstream cell-cycle transitions. In conventional *ndt80*∆ arrests, dHJs accumulate but low-level DSBs continue to form, confounding assignment of products to a defined substrate pool. More generally, meiosis endpoint analyses of joint molecule-processing mutants can be difficult to interpret because substrates themselves evolve with time and progression: intermediates can be remodelled or converted into distinct joint molecule species as cells progress through late prophase I and into division, thereby becoming accessible to, and processed by, alternative pathways. By combining pachytene arrest with acute DSB shutdown and conditional activation of late-acting pathways, we isolate a fixed cohort of pre-existing, ZMM-bound dHJ-JMs and directly compare pathway outputs on the same late intermediates.

In this substrate-defined setting, MutLγ is uniquely capable of imposing CO-biased resolution on crossover-designated dHJ-JMs. Although Mus81-Mms4 and Yen1^ON^ can process dHJ-sized JMs, they generate near-unbiased CO/NCO mixtures, arguing that CO bias is not an intrinsic property of crossover-designated intermediates revealed by endonucleolytic cleavage. Instead, CO bias emerges as a pathway-specific feature of MutLγ, consistent with yeast models in which MutLγ is activated and oriented by meiosis-specific cues and cofactors (including PCNA/RFC- and EXO1-dependent control) that are not equivalently interpreted by SSEs^11–13^.

This framework also clarifies prior reports of CO-biased Yen1^ON^ activity in standard *ndt80*∆ arrests^28^. When ongoing recombination persists, Yen1^ON^ likely acts on a broad spectrum of nascent intermediates in addition to dHJs; composite processing of these species can skew apparent CO/NCO outputs, even if cleavage of bona fide ZMM-bound dHJs is unbiased. Our data, therefore, argue against a general pre-patterning model and instead place CO bias at the level of enzyme/pathway.

Building on previous studies in yeast^11,14,15,24,42,43^, *A. thaliana*^44,45^ and mice^46^, our results support a hierarchy rather than absolute exclusivity in access to crossover-designated intermediates. ZMM-bound dHJ-JMs preferentially yield COs through MutLγ, whereas Mus81-Mms4 and Yen1^ON^ can also process these substrates efficiently but produce largely unbiased CO/NCO outputs. STR provides an additional processing route, but becomes apparent only when both MutLγ and Mus81-Mms4 are absent, and with markedly delayed kinetics. These observations would be consistent with STR gaining access to dHJ-JMs as ZMM/SC-mediated protection erodes, a transition that can be promoted by Cdc5-dependent synaptonemal complex disassembly and is delayed when JM processing is impaired^21,31^.

Because Cdc5 stimulates multiple pathways, pathway choice cannot be attributed to activation timing alone, particularly for MutLγ^12^ and Mus81-Mms4^24^. Instead, our data point to pathway-specific features that prioritise MutLγ action on ZMM-bound dHJs. One possibility is that MutLγ is pre-positioned at crossover-designated recombination nodules, an arrangement difficult to assess cytologically in budding yeast^32^ but well documented in human, mouse and plants^47–49^. In line with MutLγ being the dominant pathway in our system, *mlh3*∆ mutants exhibited the strongest delay in dHJ-JM turnover following Cdc5 induction, whereas loss of Mus81-Mms4 produced a more modest kinetic defect. This ordering suggests that, once late processing is triggered, MutLγ engages crossover-designated dHJs with higher efficiency than SSE pathways, while Mus81-Mms4, Yen1 and STR remain viable, lower-preference alternatives that can act when MutLγ is absent or when substrates deviate from the canonical ZMM-bound state.

A major outstanding question has been how the STR complex interfaces with ZMM-bound dHJs at late stages. STR is indispensable early to shape the recombination landscape, dismantling most nascent invasions to yield NCOs while enabling efficient formation of crossover-designated intermediates^14,15^, yet it also has the biochemical capacity to dismantle dHJs^50,51^. Here, conditional late depletion reveals that Top3 is required for the NCO formation observed when MutLγ and Mus81-Mms4 are absent. Conversely, STR depletion did not abolish CO bias when MutLγ was present; rather, it reduced the overall CO yield by destabilizing the pool of normal dHJ-JMs. These data argue against a strict model in which STR is obligatorily required as a direct cofactor for MutLγ-mediated resolution. Instead, STR appears to act as a competing - and potentially safeguarding - pathway that can dismantle or remodel intermediates when resolvase-mediated processing is compromised.

A central insight of this work is an Sgs1-independent function of Top3-Rmi1 in preserving the architecture of crossover-designated intermediates. Acute depletion of Top3^AID^ or Rmi1^AID^ during pachytene caused a redistribution of JM species: canonical dHJ signals decreased while aberrant high-MW joint molecules accumulated near the wells. This phenotype was Spo11-dependent, observed even when DSB shutdown was prolonged, and reproduced at a native hotspot, supporting the idea that Top3-Rmi1 suppresses aberrant JM formation from pre-established crossover-designated recombination intermediates. Notably, simultaneous depletion of Sgs1^AID^ did not suppress high-MW JM accumulation, separating this meiotic Top3-Rmi1 function from classical mitotic paradigms where Top3 counteracts Sgs1-driven toxicity^40^. These observations are consistent with a role for Top3-Rmi1 in modulating DNA topology and/or intermediate architecture in a manner that preserves resolvase-competent substrates. The biochemical properties of the high-MW JMs provide further clues. Unlike canonical dHJ-JMs, their detection was not enhanced by psoralen crosslinking, and they were sensitive to S1 nuclease, indicating enrichment for ssDNA-containing regions such as gaps, nicks, or extended single-stranded tracts. Functionally, these structures were largely refractory to MutLγ yet partially susceptible to Mus81-Mms4. This substrate selectivity offers a mechanistic explanation for why Top3-Rmi1 depletion reduces MutLγ-dependent CO yield despite leaving CO bias intact: Top3-Rmi1 prevents diversion of crossover-designated intermediates into aberrant, MutLγ-refractory species, thereby maintaining the pool of dHJ-JMs that can be productively resolved by MutLγ. In this view, Mus81-Mms4 provides a limited salvage route for at least a subset of the aberrant structures, consistent with its broader substrate range.

How might Top3-Rmi1 prevent this pathological conversion? One possibility is that Top3-Rmi1 resolves topological constraints or transient entanglements that arise as stabilized intermediates undergo branch migration^52–54^, chromatin transactions, or encounters with other DNA-based processes such as transcription. In line with this putative role, matching translocation patterns have been reported for Top3 activity as well as branch-migrating HJs towards convergent transcription sites^55,56^. Therefore, Top3-Rmi1 might specifically facilitate transcription-linked branch migration of HJs, counteracting the formation of ssDNA-rich, structurally constrained JM states that impede branch migration or resolvase access. Another possibility is that Top3-Rmi1 acts more indirectly by stabilizing factors involved in the maintenance of meiotic dHJs^57^. While we did not detect a significant perturbation of Msh5 foci, this does not exclude effects on other ZMM proteins, such as the Top3-Rmi1 interactor Mer3^58^.

The frequent alteration of SUMO/Smt3 staining upon Top3^AID^ depletion hints at an additional layer of disruption associated with aberrant joint molecule formation. While a direct role for Top3-Rmi1 in regulating SUMO dynamics cannot be excluded, our data are more consistent with an indirect effect in which high-molecular-weight JMs drive local SUMO accumulation. Such aberrant intermediates may act as platforms that recruit SUMO E3 ligases and DNA repair factors, thereby altering the SUMO landscape at sites of unresolved recombination. In line with this, recombination-associated DNA structures are established targets of the Smc5-Smc6 complex and its associated Mms21 ligase^59–61^, and persistent ssDNA within these intermediates could further promote Siz2-dependent SUMOylation of RPA at ssDNA-dsDNA interfaces^62^. In this view, altered SUMOylation is not a primary defect but a downstream consequence of impaired JM architecture, potentially reinforcing the persistence or altered processing of these structures. Dissecting the molecular composition and topology of the high-MW species, and how SUMO dynamics intersect with their formation, will be important goals for future work.

More broadly, our findings refine the conceptual framework for meiotic dHJ processing. ZMM proteins do not simply lock dHJs into a single fate; instead, they create a favoured path, MutLγ-mediated, CO-biased resolution, while alternative pathways remain poised to act when needed. Top3-Rmi1 emerges as a significant contributor in the maintenance of a subset of crossover-designated intermediates in a MutLγ-resolvable state, helping limit their conversion into aberrant structures. Such a layered hierarchy prioritizes crossover assurance to promote homologue segregation, yet preserves flexibility and backup processing routes to prevent persistent entanglements that threaten genome stability. Given the conservation of ZMM factors, MutLγ, SSEs and STR^63^, this logic is likely broadly relevant to how eukaryotes balance robust crossover formation with the capacity to avert recombination-associated chromosome segregation failure that compromises fertility. In line with this, a similar hierarchical organization may operate in plants. In *mlh3* mutants of *A. thaliana*, crossover formation is reduced by ~60%^64^, consistent with alternative nucleases, including AtMUS81^65^, acting as backup pathways. By contrast, the ~90% reduction in crossovers in *Mlh3⁻*^*/*^*⁻* mice, together with elevated BLM/Sgs1 levels^66,67^, suggests that mammals may instead rely more heavily on noncrossover pathways, such as STR/BTR, to remove unprocessed joint molecules.

## Methods

### Strain construction

All *Saccharomyces cerevisiae* strains used were SK1 derivatives and are listed in Supplementary Data 1. The following alleles were previously generated and described: *ndt80*∆^30^, *P*_*GAL1*_*-CDC5-HA3*^24^, *P*_*GPD*_*-GAL4(848)-ER*^68^, *HIS4::LEU2* locus engineered for physical analysis of recombination^10^, *REC104-FRB-HA3*^33^, *fpr1∆*^69^, *RPL13A-FKBP12*^69^, *nuc1*∆^70^, *mlh3*∆^28^, *mus81*∆^24^, *P*_*GAL1*_*-YEN1*^*ON*^*-myc18*^21^, *P*_*CLB2*_*-HA3-MMS4*^24^, *P*_*CUP1*_*-OsTIR1-F74G*^71^, *SGS1-AID-myc9*^21^, *TOP3-AID-myc9* and *RMI1-AID-myc9*^18^. Tagging *TOP3, SGS1* and *RMI1* at the C-terminus with the auxin-inducible degron (AID) was achieved by integrating the AID*−9myc cassette, amplified by PCR from plasmid pHyg-AID*−9myc^72^.

### Spore viability

Diploid cells were plated in small clumps on SPM plates (2% (w/v) potassium acetate, 20 g/L agar) and incubated for 48 h at 30 °C. A small amount of cells was taken with the end of a pipette tip and added to 50 μL 20 T zymolyase (0.1 mg/mL), then incubated for 10 min. 20 μL of the mixture was added on the side of a YPD plate and used for tetrad microdissection. The spores were grown for 48 h at 30 °C before counting. Spore viability was determined from three biological replicates (72 spores each).

### Meiotic time-courses

Meiotic time-course experiments were performed as described^73^. In brief, cells were streaked on YP-glycerol plates (20 g/L bactopeptone, 10 g/L yeast extract, 2% (v/v) glycerol, 20 g/L agar) and grown for ~48 h at 30 °C. Single colonies were selected and plated on YPD plates (20 g/L bactopeptone, 10 g/L yeast extract, 20 g/L dextrose, 20 g/L agar) as small patches, followed by ~24 h incubation at 30 °C. Cells from the patches were expanded on YPD plates as a lawn covering the entire plate and grown for 24 h at 30 °C. Depending on the amount of cells required for the experiment, cells were further expanded on YPD plates for another 24 h at 30 °C. Finally, the cells were inoculated into pre-sporulation medium (YPA; 20 g/L bactopeptone, 10 g/L yeast extract, 2% (w/v) potassium acetate) to an OD_600_ of 0.3. Cells were grown in a 10 L fermenter system^73,74^ for 14-15 h at 25 °C, reaching G1 arrest. They were washed with sporulation medium (SPM; 2% (w/v) potassium acetate), then inoculated into SPM to an OD_600_ of 3.5-4. The inoculation defines the 0 h timepoint in the experiments. Premeiotic DNA replication was followed by analysing cellular DNA content using FACS. Based on the cytological analysis of synapsis in our strains, 6-7 h after inoculation into SPM was determined as the point at which the vast majority of *ndt80*∆ cells are arrested in pachytene. DSB formation was inhibited in pachytene by anchoring away Rec104^FRB^, induced by the addition of 1 μg/mL rapamycin dissolved in DMSO. Expression of Yen1^ON^ and Cdc5^HA3^ from the *P*_*GAL1*_ promoter was induced by the addition of 4 μM β-estradiol (dissolved in methanol). To induce depletion of AID-tagged proteins, 50 μM CuSO_4_ (in dH_2_O) was added first, followed 30 min later by the addition of 5-Ph-IAA dissolved in DMSO (50 μM for Rmi1^AID^ and Sgs1^AID^ depletion, 75 μM for Top3^AID^ depletion).

### FACS analysis of DNA content

To monitor entry into the premeiotic S-phase, we followed the cellular DNA content over time. Briefly, cells from 1 mL of culture were pelleted and fixed in 70% (v/v) ice-cold ethanol. The fixed cells were washed with 1 mL of 50 mM Tris-HCl (pH 7.5), and resuspended in 0.5 mL of RNase A solution (50 mM Tris-HCl (pH 7.5), 0.4 mg/mL RNase A). Samples were incubated with RNase A for 3-4 h at 37 °C with shaking. The cells were then washed with 1 mL of FACS buffer (200 mM Tris-HCl (pH 7.5), 211 mM NaCl, 78 mM MgCl_2_) and resuspended in FACS buffer containing 55 μg/mL propidium iodide. Samples were briefly sonicated, and an aliquot was diluted 5–10-fold in 1 mL Tris-HCl (pH 7.5). DNA content was analysed on a FACSCalibur cytometer (BD Biosciences) using CellQuest Pro software (v4.0.2, BD Biosciences). Data was analysed with FlowJo software (v10.9.0, BD Biosciences)

### Protein analysis by western blotting

Proteins were extracted as previously described in ref. ^75^. In brief, 9 mL of culture was pelleted by centrifugation (3 min at 860 rcf). The cells were resuspended in 10% trichloroacetic acid and ruptured with glass beads using the FastPrep-24 5 G instrument (MP Biomedicals), running two 40 s cycles at 6 m/s. Protein precipitates were pelleted by centrifugation at 4 °C for 10 min at 21130 rcf. The pellets were resuspended in 2x NuPAGE sample buffer (Invitrogen) containing 200 mM DTT, and the acid was neutralized by adding 1 M Tris base at a 2:1 (v/v) ratio of 2x NuPAGE to Tris. Samples were boiled for 10 min at 95 °C, debris was pelleted by centrifugation for 10 min (21130 rcf), and the supernatant containing the protein extract was transferred to a new tube. Relative protein concentration in the samples was measured using the Bio-Rad Protein Assay. Samples were separated on NuPAGE 3–8% Tris-Acetate gels with NuPAGE Tris-Acetate SDS running buffer (Invitrogen) or 4–12% Bis-Tris gels with MOPS SDS running buffer (Invitrogen), and transferred to 0.45 μm PVDF membranes (Amersham Hybond, Sigma-Aldrich).

Immunoblotting was performed using the following primary antibodies: rabbit anti-Myc conjugated to HRP (1:10000, ab1326, Abcam), mouse anti-Myc (1:5000, 9E10, Cancer Research UK), mouse anti-HA.11 (1:5000, 16B12, BioLegend), mouse anti-Pgk1 (1:10000, 22C5D8, Invitrogen) and rabbit anti-Crm1 (1:5000, a gift from Karsten Weis, ETH Zürich). For detection and quantification, the following secondary antibodies were used: goat anti-mouse IgG conjugated to HRP (1:10000, P0447, Agilent), swine anti-rabbit IgG conjugated to HRP (1:10000, P0399, Agilent) and goat anti-mouse Alexa Fluor 680 (1:15000, A21057, Invitrogen). The blots were washed with PBS-T and imaged using the ChemiDoc MP Imaging System (BioRad) with Image Lab Software (v2.4.0.03, Bio-Rad). Quantification was performed in Fiji (v2.14.0/1.54 f), and images were processed using Adobe Photoshop (v25.12.0). Uncropped scans of the blots are provided in the Source data file.

### Genomic DNA isolation using guanidine/sarcosyl

Genomic DNA extraction using guanidine/sarcosyl was performed as described previously^35^. In brief, 50–100 mL of meiotic culture was pelleted and resuspended in 0.1 mg/mL trioxsalen (in 50 mM Tris, 50 mM EDTA, pH 8.0). DNA was crosslinked in the UVP Crosslinker CL-3000L (Analytik Jena) at 3600 mJ/cm^2^, while keeping cells on ice and mixing them periodically. Genomic DNA was extracted using guanidine/sarcosyl for cell lysis and phenol-chloroform for DNA purification. For analysis at the *HIS4::LEU2* locus, isolated DNA was digested overnight with XhoI (JM analysis) or double-digested with XhoI and NgoMIV (for CO/NCO analysis). DNA was quantified using the Qubit dsDNA Broad Range kit (Invitrogen).

### Genomic DNA isolation and digestion in agarose plugs

Isolation of DNA using cells embedded in agarose plugs was performed as previously described with minor modifications^76^. Briefly, 30–50 mL of meiotic culture was collected and trioxsalen-crosslinked as described above. The cell pellets were collected and stored at −80 °C. The pellets were thawed, washed with 50 mM EDTA (pH 8.0) and mixed with melted low-melting-point agarose and zymolyase solution to make ~0.5% agarose plugs ( ~90 μL each). The plugs were treated with RNase A and Proteinase K (with 1% SDS instead of 1% sarkosyl), and stored in 50 mM EDTA (pH 8.0) with 50% (w/v) glycerol. For digestion, 1/3 or 1/2 of a plug was washed 4 × 15 min in 5 mL TE buffer, then transferred to a PCR tube. Plugs were melted by incubation for 20 min at 65 °C in a thermocycler, then equilibrated at 42 °C before adding 0.5 U β-agarase and incubating for a further 45 min at 42 °C. The samples were then equilibrated at 37 °C before adding 10x CutSmart buffer and 20 U of a restriction enzyme, incubating for 2 h at 37 °C, adding 20 U more, and leaving to digest overnight. DNA was quantified using the Qubit dsDNA Broad Range kit (Invitrogen).

### S1 nuclease treatment of DNA in agarose plugs

Treatment with S1 nuclease was performed on DNA embedded in agarose plugs (as described above), as previously described^77^, with adjustments. Briefly, 2/3 of a plug was washed 4 × 15 min in 5 mL TE buffer. The 2/3 plug was then split into two 1/3 plugs. Each 1/3 plug was transferred into a separate 1.5 mL tube and equilibrated 4 × 30 min in 500 μL 1x S1 buffer (diluted from filter-sterilized 5x S1 buffer: 200 mM sodium acetate (pH 4.5), 1.5 M NaCl, 10 mM ZnSO_4_). The buffer was then replaced with fresh 200 μL of 1x S1 buffer containing 10 U S1 nuclease (Thermo Scientific), or without the nuclease for the mock treatment. Tubes were incubated on ice for 15 min and then at 37 °C for 20 min. S1 nuclease was inactivated by adding EDTA (pH 8.0) to a final concentration of 10 mM, followed by incubation on ice for 15 min. The plugs were then washed with TE, and DNA digested with restriction enzymes as described above.

### Physical analysis of recombination by Southern blotting

Physical analysis of recombination was performed as previously described^10,34,70^. In brief, for one-dimensional analysis, digested DNA (1.5–2 μg of phenol-chloroform-extracted DNA or 0.4–0.6 μg of agarose plugs DNA) was run on a 0.6% agarose gel (UltraPure agarose, Invitrogen) in 1x TBE. The gel was then stained with ethidium bromide (EtBr) to verify proper migration before transfer to a membrane. For the two-dimensional analysis, the DNA was run first in the 0.4% agarose gel (Seakem Gold, Lonza) in 1x TBE, lanes were then excised and run in the second dimension in 0.8% agarose gel (UltraPure agarose, Invitrogen) in 1xTBE with 50 μg/mL EtBr in both the gel and the buffer.

DNA was transferred from agarose gels to a Zeta-Probe GT (Bio-Rad) or GeneScreen Plus (Revvity) membranes via alkaline transfer. The membranes were then hybridized with probes for *HIS4::LEU2* and *GAT1*^10,78^. The probes were prepared and radiolabeled with [α-^32^P]dCTP using High Prime (Roche) labelling kit. After hybridization, membranes were washed and exposed to a phosphor-screen, and imaged after several days using an Amersham Typhoon phosphor imager (Cytiva) running Amersham Typhoon control software (v3.0.0.2). Different DNA species from one-dimensional blots were quantified using ImageQuant TL software (v8.1), and expressed as percentages of the total lane signal; the 0 h signal was used for background subtraction. Due to high and varied DNA shearing across phenol-chloroform-extracted DNA samples, DSB signal in those samples was first normalized by subtracting the equivalent sheared-DNA area of the lane, then using the lane with the lowest signal for further background subtraction. COs and NCOs were quantified from the CO2 and NCO2 species (XhoI + NgoMIV digest). For calculating the CO/NCO ratio following Cdc5/Yen1^ON^ induction: CO and NCO production was calculated as the increase from 8 h to 12 h (∆12h-8h) or, for AID-tagged strains, from 9 h to 13 h (∆13h-9h) from CO/NCO blots (XhoI + NgoMIV digest); the ratio was computed as ∆CO/∆NCO. JM species from two-dimensional analysis were quantified in Fiji as described in ref. ^79^. Briefly, signal intensities corresponding to DNA species were quantified, and background was subtracted. Background intensity was determined from an equivalent area of the blot lacking detectable DNA signal. Total DNA was calculated as the sum of all quantified species, including parental DNA, single-end intermediates, inter-homologue and inter-sister dHJs, and multi-chromatid joint molecules. Uncropped scans of the blots are provided in the Source data file.

### Meiotic chromosome spreads

Meiotic chromosome spreads were prepared as described in ref. ^21^. In brief, at the indicated time points, cells from 1 mL of meiotic culture were spun down (4 min at 700 rcf) and resuspended in 200 μL spheroplasting solution (2% (w/v) potassium acetate, 0.8 M sorbitol). DTT was added to a final concentration of 10 mM and incubated for 15 min at 30 °C before adding 5 μL Zymolyase 20 T solution (10 mg/mL) and digesting for ~10 min at 30 °C. The efficiency of spheroplasting was monitored by adding a 2 μL aliquot of the cells to 2 μL of 2% (w/v) sarcosyl, which lyses spheroplasted cells. When the majority of the cells in the sample were spheroplasted, 400 μL ice-cold STOP solution (0.1 M MES, 1 mM EDTA, 0.5 mM MgCl_2_, 1 M sorbitol, pH 6.4) was added to the cells to stop the digestion. Spheroplasts were spun down and (4 min at 700 rcf) resuspended in 100 μL STOP solution. 20 μL of the spheroplasts was immediately pipetted onto a clean glass slide, followed by the sequential addition of 40 μL fixative (4% (w/v) paraformaldehyde, 3.4% (w/v) sucrose), 80 μL Lipsol (1% (v/v)) and 80 μL fixative, with gentle shaking between steps. Slides were left overnight in the fume hood to dry.

Before immunostaining, the slides were washed for 15 min with 1x PBS, and then blocked by adding 200 μL blocking buffer (1% (w/v) BSA and 0.2% (w/v) gelatin in 1x PBS) and incubating the slides in a humid chamber for 20 min. For primary antibody staining, 50–80 μL of antibody mixture diluted in blocking buffer was added to the slide and covered with a coverslip, followed by a 4 h incubation at room temperature, or overnight incubation at 4 °C. Afterwards, the slides were washed three times for 5 min in 1x PBS, and then incubated with secondary antibodies. Antibodies were again diluted in blocking buffer before adding to the slides. The slides were covered with a coverslip and incubated in a humid chamber for 2–4 h at room temperature. The slides were then washed three times for 5 min in 1x PBS and mounted with ProLong Diamond Antifade Mountant (Invitrogen) containing DAPI (4’,6-diamidino-2-phenylindole) for widefield fluorescence microscopy or with ProLong Glass Antifade Mountant (Invitrogen) for STED imaging.

The following primary antibodies were used for immunostaining: rabbit anti-Zip1^80^; guinea pig anti-Rec8 (1:1000)^81^; mouse anti-Smt3 (1:500; 4F2.F5.G2, Rockland Immunochemicals); and rabbit anti-Msh5 (1:500)^82^. For widefield fluorescence microscopy, secondary antibodies conjugated to Alexa Fluor 488, Alexa Fluor 555, and Alexa Fluor 647 (1:500, Invitrogen) were used. For STED imaging, goat anti-rabbit STAR ORANGE and goat anti-guinea pig STAR RED (1:100, Abberior) were used.

A DeltaVision Ultra epifluorescence microscope (GE Healthcare), equipped with a 60x oil-immersion UPlanXApo objective (1.42 NA, working distance 0.15 mm), 100x oil-immersion UPlanSApo objective (1.4 NA, working distance 0.13 mm) and an sCMOS camera controlled by AcquireUltra software (v1.2.3) running on Linux, was used for widefield fluorescence microscopy. For STED microscopy, an Abberior STEDYCON mounted on a Zeiss Axio Imager A2 with a 100x oil-immersion alpha Plan-Apochromat objective (1.46 NA, working distance 0.11 mm) was used. Images were processed and analysed in Fiji.

### Quantification and statistical analysis

Southern and western blot quantification were done in ImageQuant and Fiji. All statistical analyses were done in Microsoft Excel (v16.0) and GraphPad Prism (v9.5.1 and v10.6.1), and statistical tests are described in figure legends.

### Reporting summary

Further information on research design is available in the Nature Portfolio Reporting Summary linked to this article.

## Supplementary information

Supplementary Information

Description of Additional Supplementary Files

Supplementary Data 1

Reporting Summary

Transparent Peer Review file

## Source data

Source Data

## Data Availability

Raw data, including all blots and microscopy images published in this study are available in the Zenodo repository (10.5281/zenodo.19556939)^83^. Source data are provided with this paper.
