# Supplementary material for: Distinct and overlapping roles of MutLγ, Mus81-Mms4, and STR in meiotic Holliday junction processing
Source: Nat Commun. 2026 Jun 2;17:7083. doi: 10.1038/s41467-026-73888-2 (PMC13392142; doi:10.1038/s41467-026-73888-2)
Supplement: Supplementary file 1 — Supplementary Information [file 41467_2026_73888_MOESM1_ESM.pdf]

SUPPLEMENTARY INFORMATION

Distinct and overlapping roles of MutLγ, Mus81-Mms4, and STR in meiotic Holliday junction processing

Authors

Lucija Orlić[1,2,3], Adrian Henggeler[1,2], Jázmin Nagy [1,2] and Joao Matos[1,2,*]

Affiliations

[1]Max Perutz Labs, Vienna BioCenter, 1030, Vienna, Austria.

[2]University of Vienna, Vienna, Austria.

[3]Vienna BioCenter PhD Program, a Doctoral School of the University of Vienna and the Medical University of Vienna, 1030 Vienna, Austria.

[*]Corresponding author. E-mail: joao.matos@maxperutzlabs.ac.at

Table of contents:

Supplementary figures 1-7

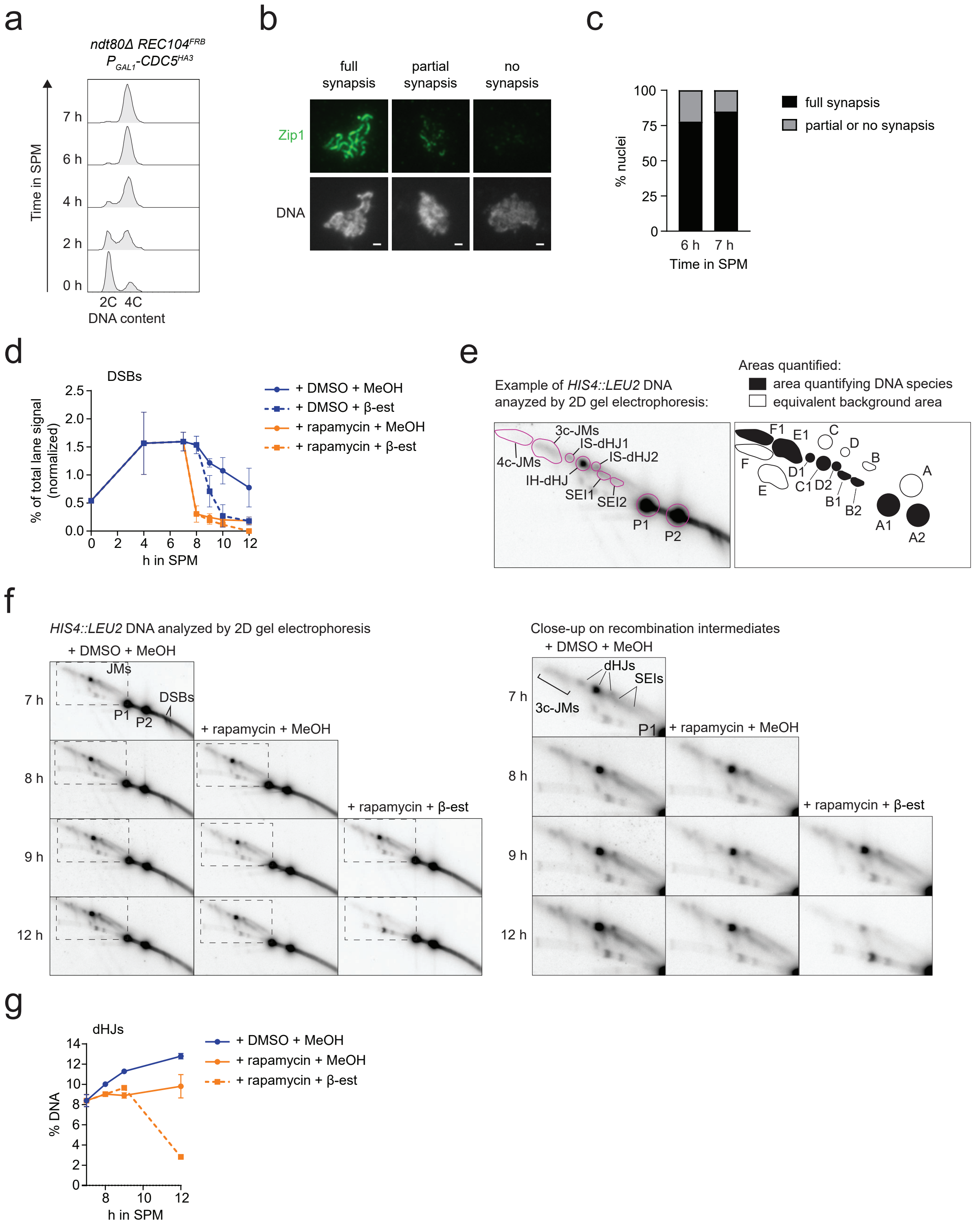
a
ndt80Δ REC104FRB
PGAL1-CDC5HA3
Time in SPM
7 h
6 h
4 h
2 h
0 h
2C 4C
DNA content
b
full synapsis
partial synapsis
no synapsis
Zip1
DNA
c
% nuclei
100
75
50
25
0
6 h
7 h
Time in SPM
full synapsis
partial or no synapsis
d
DSBs
% of total lane signal (normalized)
2.5
2.0
1.5
1.0
0.5
0.0
0 2 4 6 8 10 12
h in SPM
+ DMSO + MeOH
+ DMSO + β-est
+ rapamycin + MeOH
+ rapamycin + β-est
e
Example of HIS4::LEU2 DNA anayzed by 2D gel electrophoresis:
Areas quantified:
area quantifying DNA species
equivalent background area
4c-JMs
3c-JMs
IS-dHJ1
IS-dHJ2
IH-dHJ
SEI1
SEI2
P1
P2
F1
E1
C
D
F
B
E
D1
C1
D2
A
B1
B2
A1
A2
f
HIS4::LEU2 DNA analyzed by 2D gel electrophoresis
+ DMSO + MeOH
JMs
DSBs
P1
P2
+ rapamycin + MeOH
+ rapamycin + β-est
7 h
8 h
9 h
12 h
Close-up on recombination intermediates
+ DMSO + MeOH
dHJs
SEIs
3c-JMs
P1
+ rapamycin + MeOH
+ rapamycin + β-est
7 h
8 h
9 h
12 h
g
dHJs
% DNA
14
12
10
8
6
4
2
0
8 10 12
h in SPM
+ DMSO + MeOH
+ rapamycin + MeOH
+ rapamycin + β-est

Supplementary Figure 1. The effect of conditional DSB inhibition on recombination dynamics in pachytene-arrested *ndt80Δ* cells.

a, FACS analysis of DNA content in *ndt80Δ* $REC104^{FRB}$ P_{GAL1}-$CDC5^{HA3}$ cells. Cells were induced to undergo meiosis by transfer into sporulation medium (SPM). Representative of two independent experiments.

b, Representative widefield fluorescence images of meiotic chromosome spreads from (**a**), stained for Zip1 (green); DNA stained with DAPI (grey). Scale bars, 1 μm.

c, Quantification of Zip1 synapsis from (**b**). More than 100 cells were analyzed per timepoint, representative of two biological replicates.

d, Quantification of DSBs from Fig. 1c and a biological replicate, including β-estradiol–treated samples. Values are plotted as % total lane signal (background subtracted); mean of two independent experiments with error bars indicating range.

e, Scheme depicting the quantification methodology for various DNA species in two-dimensional (2D) gels. Signal intensity was measured from the indicated areas (black shapes), with subtracted background intensity of equivalent area on the same blot (white shapes). Levels of individual species are indicated as percentage of total defined DNA species (A-F). P1/P2 (parental DNAs); JMs (joint molecules); SEIs (single-end intermediates); dHJs (double Holliday junctions (IH, inter-homolog; IS, inter-sister)); 3c-JMs (three-chromatid joint molecules); 4c-JMs (four-chromatid joint molecules).

f, Uncropped 2D gels corresponding to Fig. 1g, together with additional 2D analyses showing the effect of Cdc5 induction (β-estradiol) on recombination intermediates at *HIS4::LEU2*. Representative of two independent experiments. DSBs, double-strand breaks.

g, Quantification of dHJs from (**f**) and a biological replicate, plotted as % total DNA signal (mean of two independent experiments; error bars, range). Source data are provided as a Source data file.

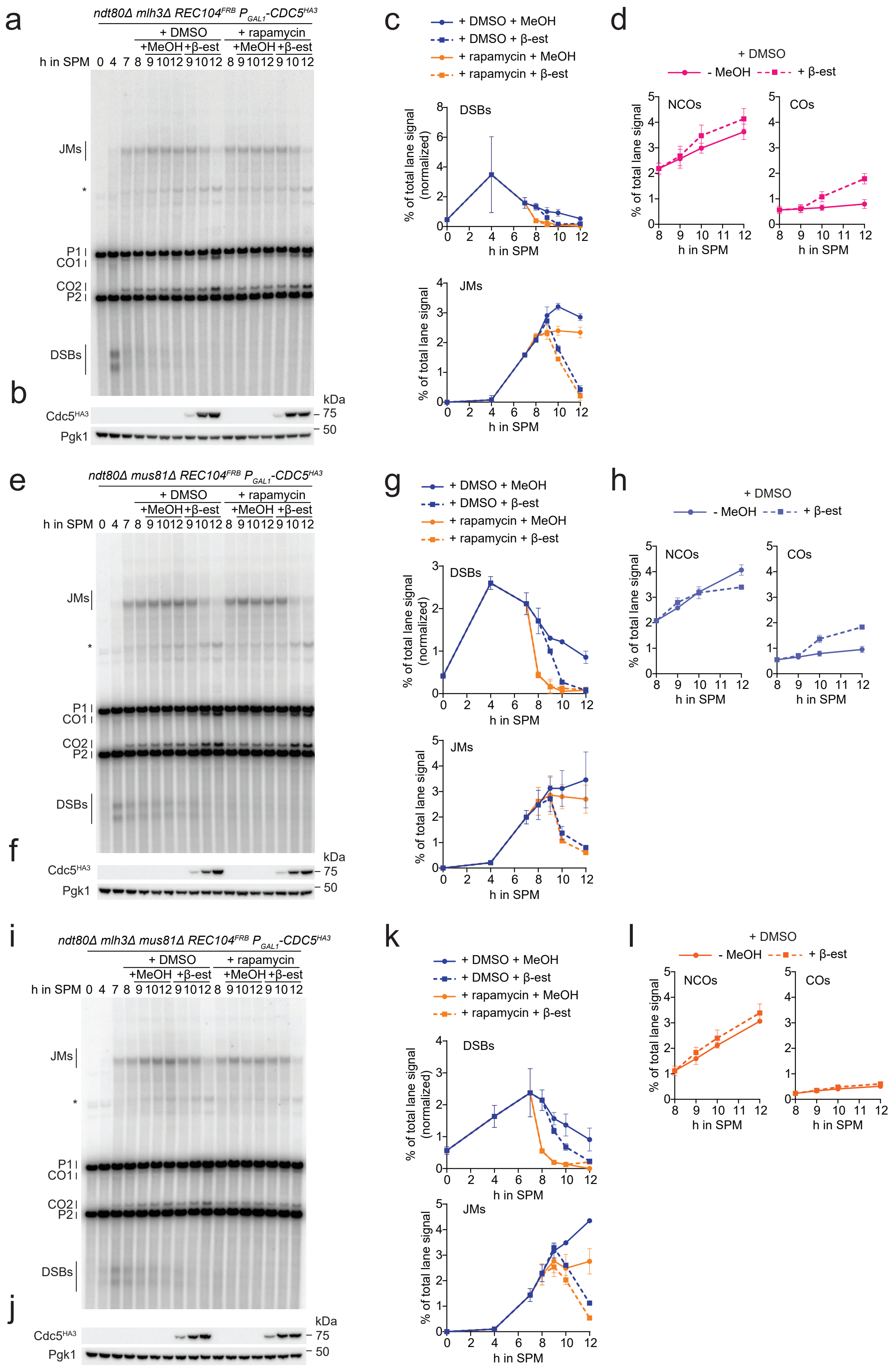

a
ndt80Δ mlh3Δ REC104FRB PGAL1-CDC5HA3
+ DMSO
+ rapamycin
+MeOH
+β-est
h in SPM
JMs
P1
CO1
CO2
P2
DSBs
b
Cdc5HA3
Pgk1
kDa
75
50
c
+ DMSO + MeOH
+ DMSO + β-est
+ rapamycin + MeOH
+ rapamycin + β-est
DSBs
% of total lane signal (normalized)
h in SPM
JMs
% of total lane signal
d
+ DMSO
- MeOH
+ β-est
NCOs
COs
e
ndt80Δ mus81Δ REC104FRB PGAL1-CDC5HA3
f
g
h
i
ndt80Δ mlh3Δ mus81Δ REC104FRB PGAL1-CDC5HA3
j
k
l

Supplementary Figure 2. DNA joint molecule dynamics and inducible joint molecule resolution in mlh3Δ, mus81Δ and mlh3Δ mus81Δ mutants

a, Physical analysis of recombination at *HIS4::LEU2* for the *mlh3Δ* experiment corresponding to Fig. 2d. Labels: dHJ-JMs (dHJ-sized joint molecules), P1/P2 (parentals), CO1/CO2 (reciprocal recombinants), DSBs (double-strand breaks). Representative of two independent experiments.
b, Western blot analysis of Cdc5 expression for samples in (**a**).
c, Quantification of DSBs and dHJ-JMs from (**a**) and a biological replicate. DSBs are plotted as % total lane signal (background subtracted); dHJ-JMs are plotted as % total lane signal. Values are mean of two independent experiments with error bars indicating range.
d, Quantification of COs and NCOs for DMSO-treated samples from Fig. 2d and a biological replicate (mean of two independent experiments; error bars, range).
e, Physical analysis of recombination at *HIS4::LEU2* for the *mus81Δ* experiment corresponding to Fig. 2g, as in (**a**). Representative of two independent experiments.
f, Western blot analysis of Cdc5 expression for samples in (**e**).
g, Quantification of DSBs and dHJ-JMs from **(e**) and a biological replicate, plotted as in (**c**) (mean of two independent experiments; error bars, range).
h, Quantification of COs and NCOs for DMSO-treated samples from Fig. 2g and a biological replicate, plotted as in (**d**) (mean of two independent experiments; error bars, range).
i, Physical analysis of recombination at *HIS4::LEU2* for the *mlh3Δ mus81Δ* experiment corresponding to Fig. 2j, as in (**a**). Representative of two independent experiments.
j, Western blot analysis of Cdc5 expression for samples in (**i**).
k, Quantification of DSBs and dHJ-JMs from (**i**) and a biological replicate, plotted as in (**c**) (mean of two independent experiments; error bars, range).
l, Quantification of COs and NCOs for DMSO-treated samples from Fig. 2j and a biological replicate, plotted as in (**d**) (mean of two independent experiments; error bars, range). Source data are provided as a Source data file.

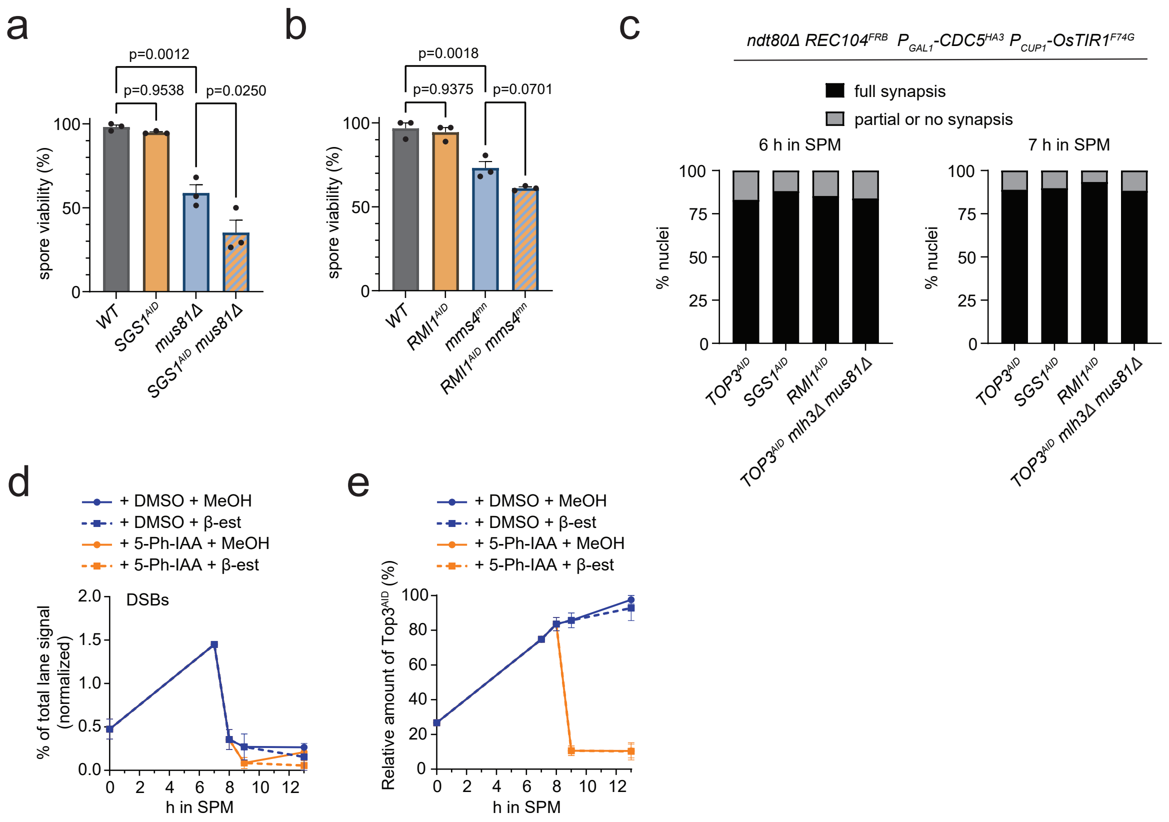

Supplementary Figure 3. Spore viability and meiotic chromosome spreads of strains carrying *$SGS1^{AID}$*, *$RMI1^{AID}$* and *$TOP3^{AID}$*

a, b, Spore viability for strains with the indicated genotypes, scored 48 h after meiosis induction on SPM plates at 30 °C. For each strain, 216 spores were analysed across three independent experiments. Data are plotted as mean ± SEM (one-way ANOVA $p < 0.0001$, followed by Tukey's multiple comparisons test). mn (meiotic null).

c, Quantification of Zip1 synapsis from indicated genotypes at 6 h and 7 h in SPM. More than 100 cells were analyzed per timepoint, representative of two biological replicates.

d, Quantification of DSBs from Fig. 3c and a biological replicate, plotted as % total lane signal (background subtracted). Values are mean of two independent experiments with error bars indicating range.

e, Relative quantification of $Top3^{AID}$ levels from Fig. 3d and a biological replicate. $Top3^{AID}$ signal was normalised to Pgk1. Values are mean of two independent experiments with error bars indicating range. Source data are provided as a Source data file.

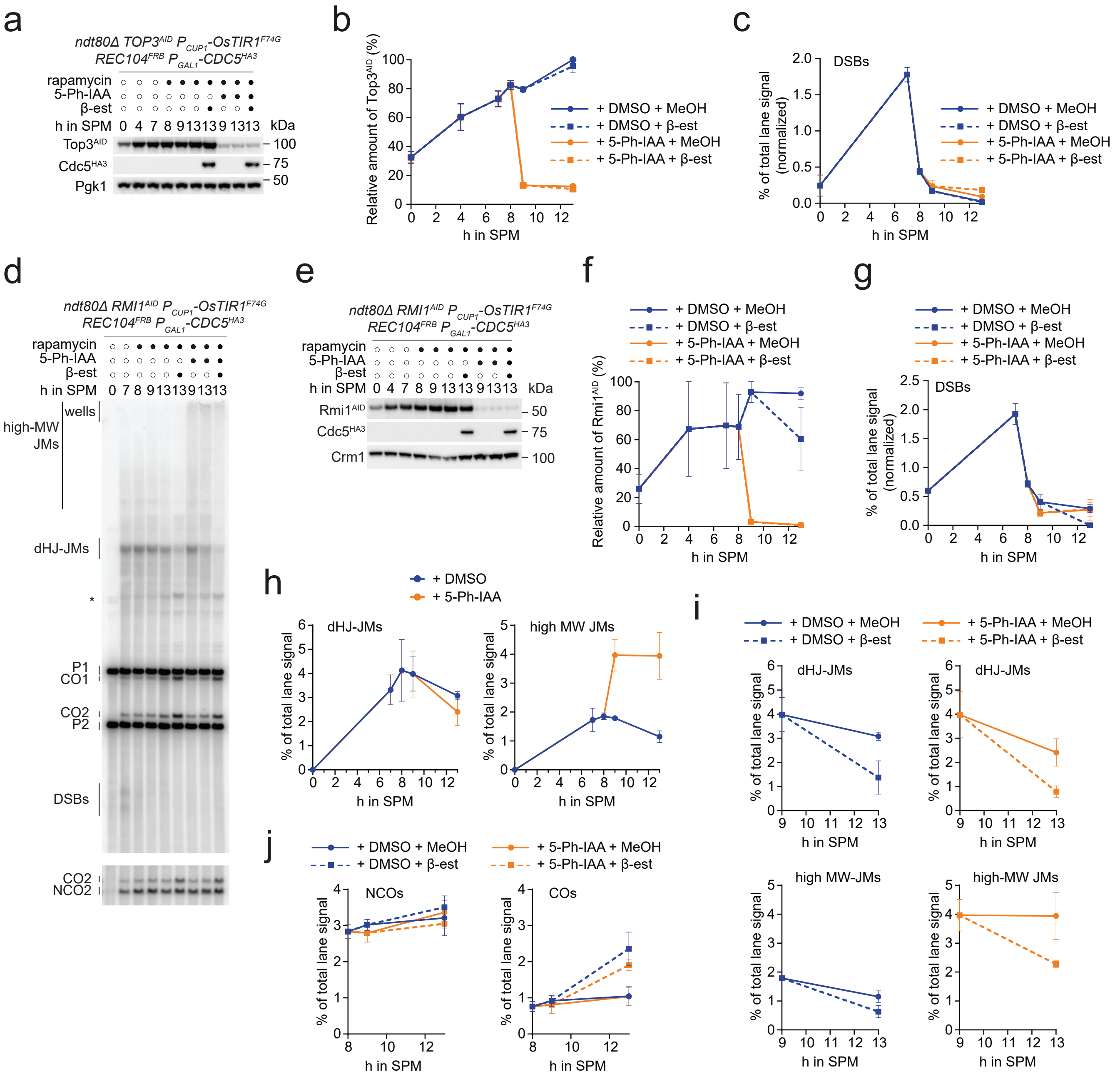
a
ndt80Δ TOP3AID PCUP1-OsTIR1F74G REC104FRB PGAL1-CDC5HA3
rapamycin
5-Ph-IAA
β-est
h in SPM 0 4 7 8 9 13 13 9 13 13
kDa
Top3AID
Cdc5HA3
Pgk1
100
75
50
b
Relative amount of Top3AID (%)
h in SPM
+ DMSO + MeOH
+ DMSO + β-est
+ 5-Ph-IAA + MeOH
+ 5-Ph-IAA + β-est
c
DSBs
% of total lane signal (normalized)
h in SPM
d
ndt80Δ RMI1AID PCUP1-OsTIR1F74G REC104FRB PGAL1-CDC5HA3
wells
high-MW JMs
dHJ-JMs
*
P1
CO1
CO2
P2
DSBs
CO2
NCO2
e
ndt80Δ RMI1AID PCUP1-OsTIR1F74G REC104FRB PGAL1-CDC5HA3
Rmi1AID
Cdc5HA3
Crm1
f
Relative amount of Rmi1AID (%)
g
DSBs
h
+ DMSO
+ 5-Ph-IAA
dHJ-JMs
high MW JMs
% of total lane signal
i
dHJ-JMs
high MW-JMs
high-MW JMs
j
NCOs
COs

Supplementary Figure 4. Rmi1 suppresses aberrant joint-molecule formation during pachytene

a, Western blot analysis for the experiment in Fig. 4a, showing Cdc5 induction and $Top3^{AID}$ depletion. Representative of two independent experiments.

b, Relative quantification of $Top3^{AID}$ levels from (**a**) and a biological replicate. $Top3^{AID}$ signal was normalised to Pgk1. Values are mean of two independent experiments with error bars indicating range.

c, Quantification of DSBs from Fig. 4a and a biological replicate, plotted as % total lane signal (background subtracted). Values are mean of two independent experiments with error bars indicating range.

d, Physical analysis of recombination at *HIS4::LEU2* in $RMI1^{AID}$ cells, performed as in Fig. 4a. Top, JM analysis (XhoI digest); bottom, CO/NCO analysis (XhoI + NgoMIV digest). dHJ-JMs (dHJ-sized joint molecules), high-MW JMs (high-molecular-weight joint molecules), asterisk (ectopic crossovers), P1/P2 (parentals), CO1/CO2 (reciprocal recombinants), DSBs (double-strand breaks), NCO2 (nonreciprocal recombinant). Representative of two independent experiments.

e, Western blot analysis for samples in (**d**), showing Cdc5 induction and $Rmi1^{AID}$ depletion. Representative of two independent experiments.

f, Relative quantification of $Rmi1^{AID}$ levels from (**e**) and a biological replicate, plotted as in (**b**). $Rmi1^{AID}$ signal was normalized to Crm1.

g, Quantification of DSBs from (**d**) and a biological replicate, plotted as in (**c**).

h, Effect of $Rmi1^{AID}$ depletion on JM abundance. dHJ-JMs (left) and high-MW JMs (right) were quantified from (**d**) and a biological replicate and plotted as % total lane signal (mean of two independent experiments; error bars, range). β-estradiol–treated samples are plotted in (**i**).

i, JM quantification from (**d**) and a biological replicate following Cdc5 induction, plotted as in (**h**).

j, Quantification of COs and NCOs from the bottom panel in (**d**) and a biological replicate, plotted as in (**h**). Source data are provided as a Source data file.

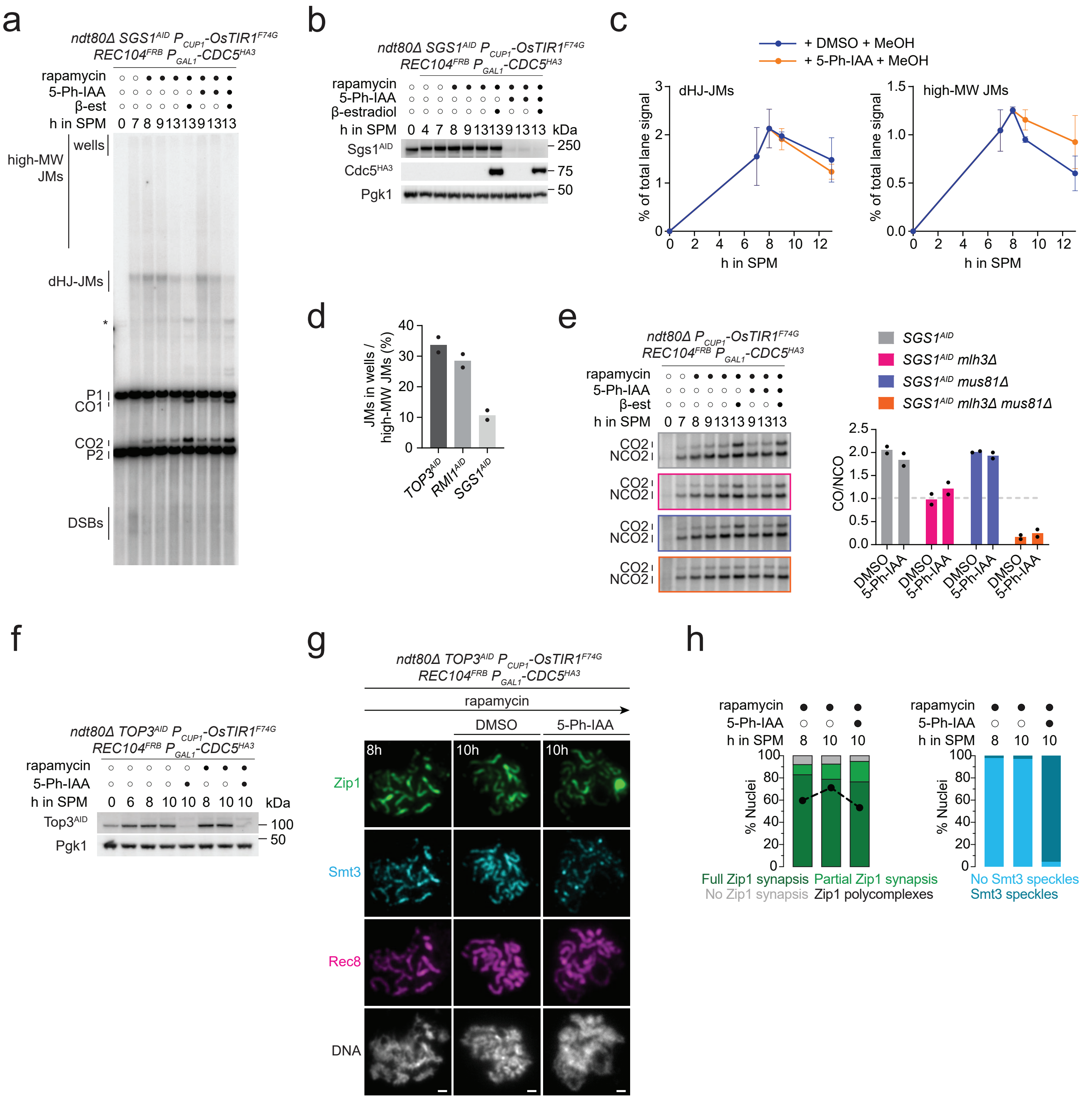
a
ndt80Δ SGS1AID PCUP1-OsTIR1F74G REC104FRB PGAL1-CDC5HA3
rapamycin
5-Ph-IAA
β-est
h in SPM 0 7 8 9 13 13 9 13 13
wells
high-MW JMs
dHJ-JMs
P1
CO1
CO2
P2
DSBs
b
ndt80Δ SGS1AID PCUP1-OsTIR1F74G REC104FRB PGAL1-CDC5HA3
rapamycin
5-Ph-IAA
β-estradiol
h in SPM 0 4 7 8 9 13 13 9 13 13 kDa
Sgs1AID
Cdc5HA3
Pgk1
250
75
50
c
+ DMSO + MeOH
+ 5-Ph-IAA + MeOH
dHJ-JMs
high-MW JMs
% of total lane signal
h in SPM
d
JMs in wells / high-MW JMs (%)
TOP3AID
RMI1AID
SGS1AID
e
ndt80Δ PCUP1-OsTIR1F74G REC104FRB PGAL1-CDC5HA3
rapamycin
5-Ph-IAA
β-est
h in SPM 0 7 8 9 13 13 9 13 13
CO2
NCO2
SGS1AID
SGS1AID mlh3Δ
SGS1AID mus81Δ
SGS1AID mlh3Δ mus81Δ
CO/NCO
DMSO
5-Ph-IAA
f
ndt80Δ TOP3AID PCUP1-OsTIR1F74G REC104FRB PGAL1-CDC5HA3
rapamycin
5-Ph-IAA
h in SPM 0 6 8 10 10 8 10 10 kDa
Top3AID
Pgk1
100
50
g
ndt80Δ TOP3AID PCUP1-OsTIR1F74G REC104FRB PGAL1-CDC5HA3
rapamycin
DMSO
5-Ph-IAA
8h
10h
10h
Zip1
Smt3
Rec8
DNA
h
rapamycin
5-Ph-IAA
h in SPM 8 10 10
% Nuclei
Full Zip1 synapsis
Partial Zip1 synapsis
No Zip1 synapsis
Zip1 polycomplexes
No Smt3 speckles
Smt3 speckles

Supplementary Figure 5. ***SGS1^AID^*** **does not phenocopy** ***TOP3^AID^*** **or** ***RMI1^AID^***

a, Physical analysis of recombination intermediates at *HIS4::LEU2* in *SGS1^AID^* cells, performed as in Fig. 4a. XhoI digest for JM analysis. dHJ-JMs (dHJ-sized joint molecules), high-MW JMs (high-molecular-weight joint molecules), asterisk (ectopic crossovers), P1/P2 (parentals), CO1/CO2 (reciprocal recombinants), DSBs (double-strand breaks). Representative of two independent experiments.
b, Western blot analysis for samples in (**a**), showing Cdc5 induction and Sgs1^{AID} depletion. Representative of two independent experiments.
c, Quantification of dHJ-JMs (left) and high-MW JMs (right) from (**a**) and a biological replicate, plotted as % total lane signal. Values are mean of two independent experiments with error bars indicating range.
d, Share of JM signal retained in wells in overall high-MW JM signal, plotted as % of high MW-JM signal for Top3^{AID}-depleted cells (from Fig. 4a), Rmi1^{AID}-depleted cells (from Supplementary Fig. 4d) and Sgs1^{AID}-depleted cells (from (**a**)), including independent replicates for each. Values are mean of two independent experiments.
e, CO/NCO ratios following Cdc5 induction (9–13 h in SPM). COs and NCOs were quantified from blots on the left (and biological replicates) and used to calculate CO/NCO ratios for the indicated genotypes and conditions. Values are mean of two independent experiments. CO/NCO > 1, crossover-biased; ~1, unbiased; <1, noncrossover-biased.
f, Western blot analysis of Top3^{AID} depletion for samples used in Fig. 4h.
g, Representative widefield fluorescence images of meiotic chromosome spreads from Fig. 4h, stained for Zip1 (green), Smt3 (cyan) and Rec8 (magenta); DNA stained with DAPI (grey). Scale bars, 1 µm.
h, Quantification of synapsis (left) and nuclei with Smt3 speckles (right) from spreads in (**g**). Synapsis scoring criteria are as described in [21]. n = 250 nuclei were analysed per condition. Zip1 polycomplex frequency is indicated by the dotted line. Smt3 speckles correspond to discrete foci of increased Smt3 intensity superimposed on the elongated Smt3 structures in 5-Ph-IAA-treated samples. Source data are provided as a Source data file.

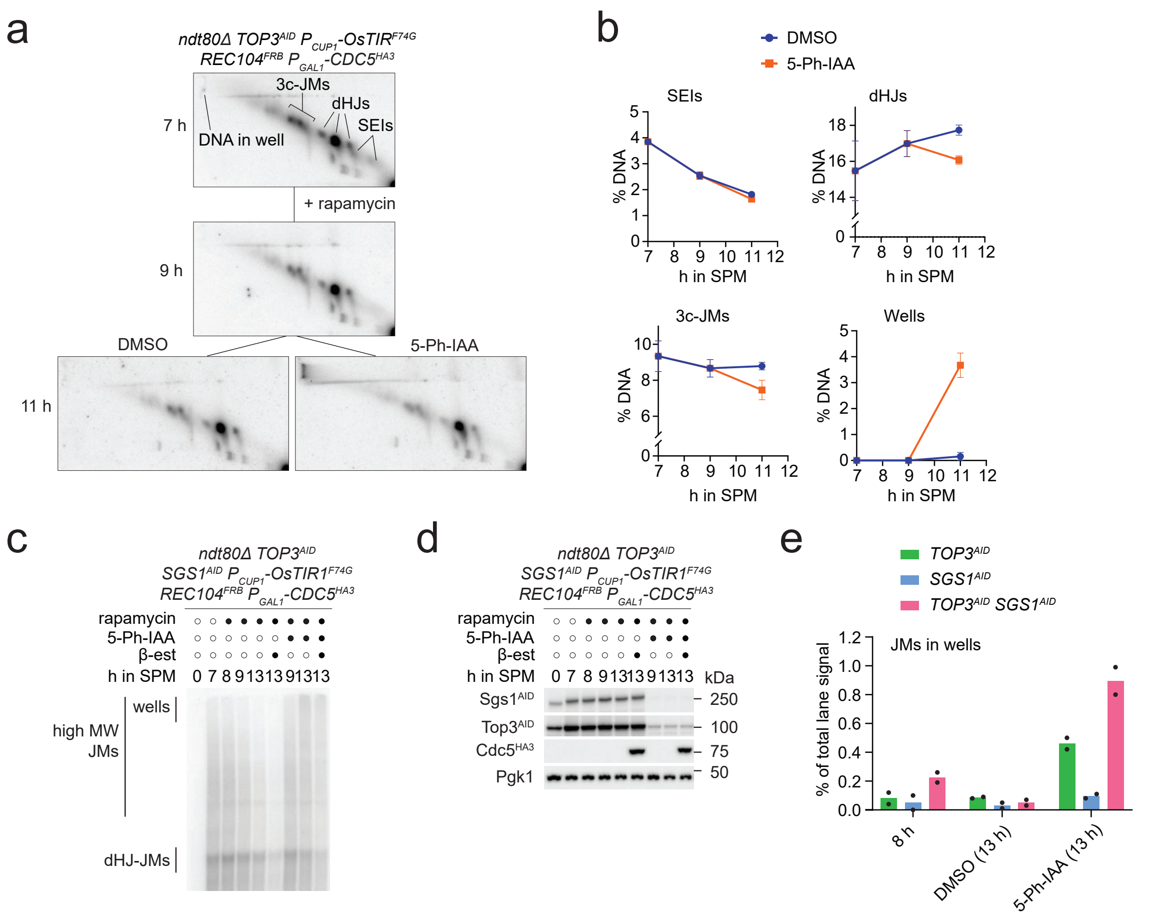

Supplementary Figure 6. Aberrant joint molecules formed due to pachytene depletion of Top3^{AID} are Sgs1-independent

a, Two-dimensional analysis of recombination intermediates at *HIS4::LEU2* from *SPO11* cells from the experiment in Fig. 5a. Images are representative of two independent experiments. SEIs (single-end intermediates), dHJs (double Holliday junctions; centre, inter-homolog; left/right, inter-sister), 3c-JMs (joint molecules involving three chromatids).

b, Quantification of recombination intermediates from (**a**) and a biological replicate, plotted as % total DNA signal. Values are means of two independent experiments with error bars indicating range.

c, Physical analysis of recombination at *HIS4::LEU2* from *TOP3^{AID} SGS1^{AID}* cells, performed as in Fig. 3b. dHJ-JMs (dHJ-sized joint molecules), high-MW JMs (high-molecular-weight joint molecules). Images are representative of two independent experiments.

d, Western blot analysis for samples in (**c**), showing depletion of Sgs1^{AID} and Top3^{AID}, and induction of Cdc5.

e, Quantification of JM signal retained in wells for the indicated genotypes and conditions, plotted as % total lane signal. Data were quantified from (**c**), Fig. 4a, Supplementary Fig. 5a, and biological replicates. Values are means of two independent experiments. Source data are provided as a Source data file.

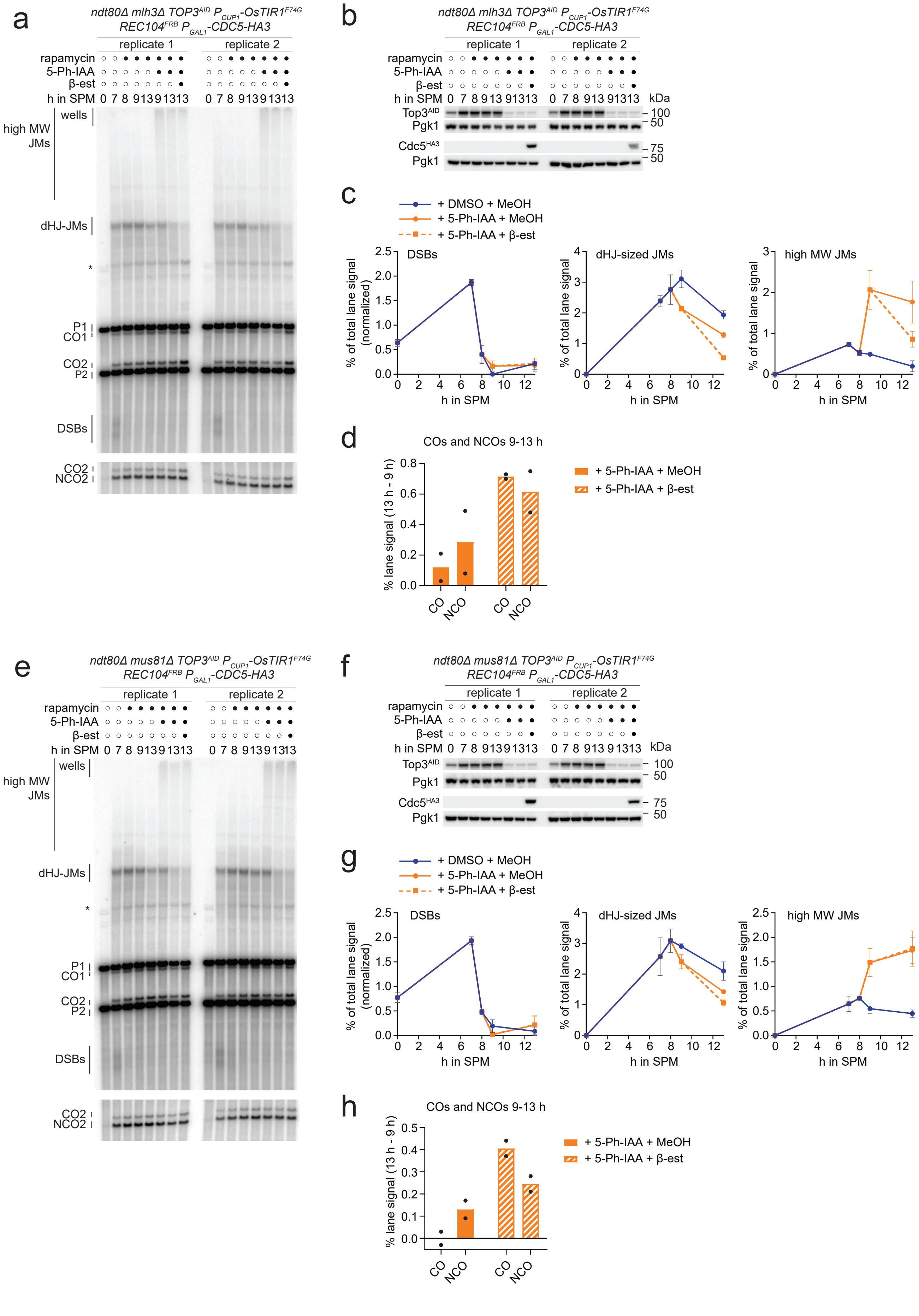
a
ndt80Δ mlh3Δ TOP3AID PCUP1-OsTIR1F74G
REC104FRB PGAL1-CDC5-HA3
replicate 1
replicate 2
rapamycin
5-Ph-IAA
β-est
h in SPM 0 7 8 9 13 9 13 13
wells
high MW JMs
dHJ-JMs
*
P1
CO1
CO2
P2
DSBs
CO2
NCO2
b
ndt80Δ mlh3Δ TOP3AID PCUP1-OsTIR1F74G
REC104FRB PGAL1-CDC5-HA3
kDa
Top3AID
Pgk1
Cdc5HA3
100
50
75
c
+ DMSO + MeOH
+ 5-Ph-IAA + MeOH
+ 5-Ph-IAA + β-est
DSBs
dHJ-sized JMs
high MW JMs
% of total lane signal (normalized)
% of total lane signal
h in SPM
d
COs and NCOs 9-13 h
% lane signal (13 h - 9 h)
CO
NCO
e
ndt80Δ mus81Δ TOP3AID PCUP1-OsTIR1F74G
REC104FRB PGAL1-CDC5-HA3
f
ndt80Δ mus81Δ TOP3AID PCUP1-OsTIR1F74G
REC104FRB PGAL1-CDC5-HA3
g
h
COs and NCOs 9-13 h

Supplementary Figure 7. Mus81-Mms4 can partially process aberrant JMs formed upon Top3^{AID} depletion.

a, Full images of physical analysis of recombination at *HIS4::LEU2* from *mlh3Δ TOP3^{AID}* cells corresponding to Fig. 5k, l. The top panel shows JM analysis (XhoI digest) and the bottom panel shows CO/NCO analysis (XhoI + NgoMIV digest). dHJ-JMs (dHJ-sized joint molecules), high-MW JMs (high-molecular-weight joint molecules), asterisk (ectopic crossovers), P1/P2 (parentals), CO1/CO2 (reciprocal recombinants), DSBs (double-strand breaks), NCO2 (nonreciprocal recombinant). Two independent experiments are shown.
b, Western blot analysis for the samples in (**a**), showing Cdc5 induction and depletion of Top3^{AID}.
c, Quantification of DSBs and JMs from (**a**, top panel) and the corresponding biological replicate. DSBs are plotted as % total lane signal (background-subtracted). JMs are plotted as % total lane signal. Values are means of two independent experiments with error bars indicating range.
d, CO and NCO quantification from 5-Ph-IAA-treated samples in (**a**, bottom panel). Values are means of two independent experiments.
e, Full images of physical analysis of recombination at *HIS4::LEU2* from *mus81Δ TOP3^{AID}* cells corresponding to Fig. 5m, n, presented as in (**a**). Two independent experiments are shown.
f, Western blot analysis for the samples in (**e**), showing Cdc5 induction and depletion of Top3^{AID}.
g, Quantification of DSBs and JMs from (**e**, top panel), presented as in (**c**).
h, CO and NCO quantification from 5-Ph-IAA-treated samples in (**e**, bottom panel), presented as in (**d**). Source data are provided as a Source data file.
