# Supplementary material for: Distinct and overlapping roles of MutLγ, Mus81-Mms4, and STR in meiotic Holliday junction processing
Source: Nat Commun. 2026 Jun 2;17:7083. doi: 10.1038/s41467-026-73888-2 (PMC13392142; doi:10.1038/s41467-026-73888-2)
Supplement: Supplementary file 2 — Description of Additional Supplementary Files [file 41467_2026_73888_MOESM2_ESM.pdf]

Description of Additional Supplementary Files

File name: Supplementary Data 1

Description: A list of budding yeast strains used in this study
