# Supplementary material for: Distinct and overlapping roles of MutLγ, Mus81-Mms4, and STR in meiotic Holliday junction processing
Source: Nat Commun. 2026 Jun 2;17:7083. doi: 10.1038/s41467-026-73888-2 (PMC13392142; doi:10.1038/s41467-026-73888-2)
Supplement: Supplementary file 4 — Reporting Summary [file 41467_2026_73888_MOESM4_ESM.pdf]

nature portfolio

Corresponding author(s): Joao Matos

Last updated by author(s): May 8, 2026

Reporting Summary

Nature Portfolio wishes to improve the reproducibility of the work that we publish. This form provides structure for consistency and transparency in reporting. For further information on Nature Portfolio policies, see our Editorial Policies and the Editorial Policy Checklist.

Statistics

For all statistical analyses, confirm that the following items are present in the figure legend, table legend, main text, or Methods section.

n/a	Confirmed	
☐	☒	The exact sample size (*n*) for each experimental group/condition, given as a discrete number and unit of measurement
☐	☒	A statement on whether measurements were taken from distinct samples or whether the same sample was measured repeatedly
☐	☒	The statistical test(s) used AND whether they are one- or two-sided<br>*Only common tests should be described solely by name; describe more complex techniques in the Methods section.*
☒	☐	A description of all covariates tested
☐	☒	A description of any assumptions or corrections, such as tests of normality and adjustment for multiple comparisons
☐	☒	A full description of the statistical parameters including central tendency (e.g. means) or other basic estimates (e.g. regression coefficient) AND variation (e.g. standard deviation) or associated estimates of uncertainty (e.g. confidence intervals)
☐	☒	For null hypothesis testing, the test statistic (e.g. *F*, *t*, *r*) with confidence intervals, effect sizes, degrees of freedom and *P* value noted<br>*Give P values as exact values whenever suitable.*
☒	☐	For Bayesian analysis, information on the choice of priors and Markov chain Monte Carlo settings
☒	☐	For hierarchical and complex designs, identification of the appropriate level for tests and full reporting of outcomes
☒	☐	Estimates of effect sizes (e.g. Cohen's *d*, Pearson's *r*), indicating how they were calculated

Our web collection on statistics for biologists contains articles on many of the points above.

Software and code

Policy information about availability of computer code

Data collection	Deltavision Ultra (GE Healthcare): AcquireUltra (version 1.2.3)<br>Abberior STEDYCON: STEDYCON smart control (version 7.1.53);<br>BD FACSCalibur: BD CellQuest Pro (4.0.2);<br>ChemiDoc MP Imaging System (Bio-Rad): Image Lab Software (2.4.0.03);<br>Amersham Typhoon phosphor imager (Cytiva): Amersham Typhoon control software (3.0.0.2)
Data analysis	No custom software or code was used.<br>Cytological analyses were performed using Fiji (version 2.14.0/1.54f). Western blots were quantified in Fiji and prepared for presentation in Fiji and Adobe Photoshop (version 25.12.0). FACS data were analyzed using FlowJo (version 10.9.0). Southern blots were quantified with ImageQuant TL (version 8.1) or Fiji and adapted for presentation in Fiji. Graphs were generated, and all statistical analyses were performed in GraphPad Prism (version 9.5.1 and version 10.6.1), Microsoft Excel for Mac (version 16.87) or Windows (version 16.0). Figures and schemes were assembled in Adobe Illustrator (version 29.8.1, 2025).

For manuscripts utilizing custom algorithms or software that are central to the research but not yet described in published literature, software must be made available to editors and reviewers. We strongly encourage code deposition in a community repository (e.g. GitHub). See the Nature Portfolio guidelines for submitting code & software for further information.

Data

Policy information about availability of data

All manuscripts must include a data availability statement. This statement should provide the following information, where applicable:

- Accession codes, unique identifiers, or web links for publicly available datasets
- A description of any restrictions on data availability
- For clinical datasets or third party data, please ensure that the statement adheres to our policy

All relevant data generated or analyzed during this study are included in this article and its Supplementary Information file. Biological materials are available from the corresponding author.

Research involving human participants, their data, or biological material

Policy information about studies with human participants or human data. See also policy information about sex, gender (identity/presentation), and sexual orientation and race, ethnicity and racism.

Reporting on sex and gender	The study did not involve human participants, their data, or biological material.
Reporting on race, ethnicity, or other socially relevant groupings	The study did not involve human participants, their data, or biological material.
Population characteristics	The study did not involve human participants, their data, or biological material.
Recruitment	The study did not involve human participants, their data, or biological material.
Ethics oversight	The study did not involve human participants, their data, or biological material.

Note that full information on the approval of the study protocol must also be provided in the manuscript.

Field-specific reporting

Please select the one below that is the best fit for your research. If you are not sure, read the appropriate sections before making your selection.

☒ Life sciences ☐ Behavioural & social sciences ☐ Ecological, evolutionary & environmental sciences

For a reference copy of the document with all sections, see nature.com/documents/nr-reporting-summary-flat.pdf

Life sciences study design

All studies must disclose on these points even when the disclosure is negative.

Sample size	All sample sizes and the number of biological replicates for each experiment are reported in the figure legends. No statistical methods were used to predetermine sample size. For cytological analyses, sample sizes were guided by preliminary experiments that helped establish an appropriate sample size for the purpose of this study. For Southern and Western blot assays, the number of experiments performed followed standard practices in the field.
Data exclusions	No data were excluded from the analyses.
Replication	Experimental findings were confirmed by performing multiple biological replicates as indicated in the figure legends.
Randomization	Not applicable. Experiments involved comparisons between control (wild type or other appropriate controls) and mutant or treated yeast cells; randomization was therefore neither necessary nor appropriate.
Blinding	Blinding was not used during data collection and analysis. All results involved side-by-side comparisons of mutant or treated yeast cells with appropriate controls.

Reporting for specific materials, systems and methods

We require information from authors about some types of materials, experimental systems and methods used in many studies. Here, indicate whether each material, system or method listed is relevant to your study. If you are not sure if a list item applies to your research, read the appropriate section before selecting a response.

Materials & experimental systems

n/a	Involved in the study
☐	☒ Antibodies
☐	☒ Eukaryotic cell lines
☒	☐ Palaeontology and archaeology
☒	☐ Animals and other organisms
☒	☐ Clinical data
☒	☐ Dual use research of concern
☒	☐ Plants

Methods

n/a	Involved in the study
☒	☐ ChIP-seq
☒	☐ Flow cytometry
☒	☐ MRI-based neuroimaging

Antibodies

Antibodies used	Primary antibodies for Western blotting were: rabbit anti-Myc conjugated to HRP (1:10000, ab1326, Abcam), rabbit anti-Crm1 (1:5000, gift from K. Weis), mouse anti-Myc (1:5000, 9E10, Cancer Research UK), mouse anti-Pgk1 (1:10000, 22C5D8, Invitrogen), and mouse anti-HA.11 (1:5000, 16B12, BioLegend).<br><br>Secondary antibodies for Western blotting included goat anti-mouse IgG conjugated to HRP (1:10000, P0447, Agilent), swine anti-rabbit IgG conjugated to HRP (1:10000, P0399, Agilent) and goat anti-mouse IgG conjugated to Alexa Fluor 680 (1:15000, A21057, Invitrogen).<br><br>Primary antibodies for cytological analysis included: rabbit anti-Zip1 (1:1000, Grigaitis et al., 2020), guinea pig anti-Rec8 (1:1000, Bommi et al., 2019), rabbit anti-Msh5 (1:500, Shinohara et al., 2008) and mouse anti-Smt3/SUMO (1:500, 4F2.F5.G2, Rockland Immunochemicals).<br><br>Secondary antibodies for cytology were goat or donkey antibodies conjugated to Alexa Fluor 488, Alexa Fluor 555, and Alexa Fluor 647 (1:500, Invitrogen). For STED microscopy, secondary antibodies included goat anti-rabbit STAR ORANGE (Abberior) and goat anti-guinea pig STAR RED (1:100, Abberior).
Validation	All home-made antibodies were characterized and validated with appropriate controls for both Western blotting and cytological analyses in previous publications, as cited above. The specificity of anti-HA antibody (16B12, BioLegend) was validated by detection of bands at the appropriate molecular weights in Western blotting upon induced expression of tagged proteins (Cdc5-HA3 in Fig. 1b), compared to non-induced controls. The specificity of anti-Myc antibodies was validated by the reduction of detected signal upon induced depletion of tagged proteins (Top3-AID-myc9 in Fig. 3d,), compared to non-depleted controls.<br>The specificity of anti-Crm1 (gift from K. Weis) and anti-Pgk1 (22C5D8, Invitrogen) was validated by detection of bands at the appropriate molecular weights in Western blotting. The specificity of anti-Smt3/SUMO (4F2.F5.G2, Rockland Immunochemicals) for immunofluorescence was confirmed in a previous publication (Henggeler et al., 2025).

Eukaryotic cell lines

Policy information about cell lines and Sex and Gender in Research

Cell line source(s)	We used budding yeast strains, all derivatives of SK1. Detailed genotypes are provided in Supplementary Data 1.
Authentication	n/a
Mycoplasma contamination	n/a
Commonly misidentified lines<br>(See ICLAC register)	n/a

Plants

Seed stocks	The study did not involve plant seeds.
Novel plant genotypes	The study did not involve plant.
Authentication	The study did not involve plants.
