# Supplementary material for: Distinct and overlapping roles of MutLγ, Mus81-Mms4, and STR in meiotic Holliday junction processing
Source: Nat Commun. 2026 Jun 2;17:7083. doi: 10.1038/s41467-026-73888-2 (PMC13392142; doi:10.1038/s41467-026-73888-2)
Supplement: Supplementary file 5 — Transparent Peer Review file [file 41467_2026_73888_MOESM5_ESM.pdf]

nature portfolio

Peer Review File

Distinct and overlapping roles of MutLγ, Mus81-Mms4, and STR in meiotic Holliday junction processing

Corresponding Author: Professor Joao Matos

This file contains all reviewer reports in order by version, followed by all author rebuttals in order by version.

Version 0:

Reviewer comments:

Reviewer #1

(Remarks to the Author)
This study addresses the regulation and resolution of meiotic recombination intermediates by combining an ndt80Δ arrest with rapamycin inducible anchor away depletion of Rec104. By temporally and conditionally controlling DSB formation, the authors achieve an unusually tight experimental window to interrogate the fate of ZMM-designated double Holliday junctions (dHJs) that give rise to a subset of crossover biased recombination events. From the outset, the sheer volume of data is striking and conveys the enormous experimental investment underlying the work, with time consuming, meticulously implemented assays and cleverly engineered genetic tools deployed throughout.

The work is distinguished by the elegant use of the anchor away system to acutely inactivate Rec104, providing a level of regulatory precision that represents the paper's key conceptual and technical advance. A comprehensive and rigorously controlled experimental framework—centered on one and two dimensional agarose gel analyses by southern blotting monitoring break formation, repair progression, and resolution outcomes—is of exceptional technical quality. Overall, the study is meticulously executed, conceptually coherent, and experimentally beautiful, leaving little to critique in terms of design or controls, and stands as an exemplary contribution to the field of meiotic recombination.

I have no major concerns. The following comments are suggestions to improve clarity and discussion.

1- For readers familiar with two dimensional agarose gel analyses, the beauty of the gels stands out as an extremely well crafted experiment. I will nonetheless ask for a little more detail on how the quantification of the different Holliday junction species was performed on the two dimensional gels. The authors appropriately refer to reference 62 for the quantification procedure. However, to facilitate reading of the Results—for example Figure 1 (panels g and h)—and to better understand where the percentage of dHJs comes from, it would be useful to clarify what is defined as total DNA signal (all species and smearing, or only defined species). This could be visually indicated in Expanded Figure 1, where the complete gel with P1 and P2 spots is shown, for example by adding a simple schematic and a clarification in the figure legend.
This comment is not meant to question the validity of the quantifications, but reflects a genuine interest in making the analysis easier to follow directly from the paper, without the need to go deeper into referenced methods.

2- This reviewer shares with the authors the view that one of the most original insights of this work is the identification of an Sgs1-independent function of Top3-Rmi1 in preserving the architecture of crossover designated intermediates. In the Discussion, the authors link this observation to changes in the accumulation of SUMOylated substrates. I would suggest expanding this line of discussion by considering some known properties of SUMO ligases in substrate recognition during DNA repair. Through their SAP domains, ligases such as Siz2 can scan dsDNA in search of substrates, and as described for RPA sumoylation, this can orient local SUMOylation at ssDNA–dsDNA boundaries. Top3, by altering the transitions of joint molecule intermediates, may influence the formation of "landing paths" for SUMO ligases or the availability of substrates at the boundaries of these remodeled species with increased ssDNA.

I think that identifying the positions of SUMO ligases, rather than measuring only global SUMOylation, could shed light on the mechanisms underlying the Top3 depletion phenotypes, and represents an interesting direction for future investigation.

Reviewer #2

(Remarks to the Author)
This is an elegantly executed study that takes advantage of a very sophisticated system developed by the Matos lab to monitor pre-existing double-Holliday junction (dHJ) structures in yeast meiosis. A key feature of this system is that it removes potential complications associated with the continued initiation of recombination events in a mutant background (ndt80) that stabilizes dHJ structures in pachytene. The current work takes advantage of clever methods to prevent the induction of new recombination events in pachytene arrested cells and uses a construct to initiate dHJ resolution through induction of Cdc5 kinase. It also uses constructs to selectively degrade components of Sgs1-Top3-Rmi1, which were shown previously to act in multiple steps in meiotic recombination, including the prevention of aberrant DNA joint molecules and the promoting the formation of crossover-designated recombination intermediates. The goal of this work is to answer two questions posed by the authors in the abstract: 1. How are dHJs formed in the crossover-specific pathway directed toward resolution by the MutLgamma resolvase? 2. Is there a previously unknown role for the Sgs1-Top3-Rmi1 complex in maintaining the architecture of the dHJ in the crossover specific pathway?

The extremely detailed and sophisticated experiments presented in the manuscript are consistent with the authors conclusions. I agree with the authors that it supports “a hierarchy in dHJ processing, one that favors MutLgamma-directed crossovers while preserving alternative resolution routes to safeguard chromosome segregation.” The work also resolves a curiosity in the field in terms of whether ectopic expression of an activated Yen1 resolvase can lead to biased resolution of dHJs to form crossovers; the new work indicates that this is not the case. Lastly, it provides new information on an independent role for Top3-Rmi1 in preventing the conversion of dHJs in the crossover pathway from being processed into aberrant intermediates that cannot be acted upon by the MutLgamma resolvase. The comments listed below are aimed at improving the clarity of the manuscript.

1. The idea of a hierarchy in dHJ processing has been surmised for some time, beginning with genetic studies (some examples include de los Santos et al., Genetics 164:81; Argueso et al., Genetics 168:1805). I think that it would be appropriate to recognize these earlier studies. This doesn’t take away from how the current studies provide mechanistic evidence for a hierarchy argument. With this said I could see the value in speculating how the submitted work could be relevant to understanding meiotic recombination in higher eukaryotes. In baker’s yeast the MLH3 knockout only reduces crossing over by about 50% whereas in mouse MSH4/5 and MLH1/3 knockouts cause much more severe defects in crossing over. Also, in yeast the number of MutSgamma foci in pachytene is roughly equivalent to the ultimate number of meiotic crossovers; in mouse the number of Msh4-Msh5 foci is paired down significantly to approach the number of MutLgamma foci.

2. The anchor away system performed in the ntd80null background is extremely powerful-it would be worth briefly stating how the kinetics of this system (e.g. the half-lives of DSBs, JMs, dHJs in the +rapamycin, + estradiol protocol presented in Fig. 1A) compares to the published half-lives in a standard wild-type SK1 time course that examines intermediates at the HIS4LEU2 hotspot.

3. Figure 3F. A control for active Top3 (+rapamycin, -5-phenol-IAA, +beta-estradiol) is not included. I think that it’s important to include such a control within the same experiment to support the argument that the “NCOs that still arise in the mlh3null mus81null background depend on Top3….”

4. Line 98. The work of Sanchez et al. is cited in the manuscript but it’s worth adding a bit of context. Sanchez identified a non-canonical physical interaction between Exo1 and MutLgamma that is critical for MutLgamma activation, though the target of Cdc5 phosphorylation is unclear-it does not appear to be either Mlh1 or Mlh3.

5. Line 132 The FACS analysis only indicates that DNA premeiotic replication has been completed; it doesn’t provide a direct measure that cells had reached pachytene at the time when Rec104 is anchored away. This sentence should be clarified. Can the authors provide more direct evidence that the various strains used in this study are in pachytene at T=7 hrs?

6. Line 160. Figure 1n. I think that the authors should spend a bit more effort defining the CO/NCO calculation. The calculation is a critical aspect of the paper that provides, at least for me, new evidence, through Rec104 anchor-away and Cdc5 activation experiments, that MutLgamma acts in a biased CO resolution pathway. The fact that this calculation involves measuring changes in CO and NCO levels after CDC5 activation is something that may not be obvious to many readers. This method essentially removes the ~80% of NCOs and the ~20% of COs that appear before CDC5 activation.

As an aside, it’s interesting that NCOs rise at a faster rate when Rec104 is not anchored away and that this results in a CO/NCO ratio of ~1. The authors don’t really comment on the continuous rise of NCOs-could this reflect a homeostasis mechanism where COs have already been designated and the increasing DSBs are shunted primarily to a NCO pathway?

7. Beginning on line 475. The text positions Top3 as a “key architectural safeguard” but does not discuss how other Top3 interactors (e.g. Mer3, Altmannova et al., NAR 51:4363) may impact the protection of CO-designated intermediates. While work in this study suggests that Sgs1 does not play the safeguard role proposed for Top3, it’s worth noting that Chang et al. (EMBO J. 24:2024) reported that Sgs1 levels decrease in the absence of Top3, so other proteins could be impacted by the loss of Top3 function. There could be value of toning down the arguments presented in the Discussion to be open to a more complex mechanism.

Reviewer #3

(Remarks to the Author)
Crossing over between homologous chromosomes is needed in most species to promote accurate chromosome segregation at the first meiotic division. The field has learned much in the past couple of decades about the mechanisms that carry out crossover formation and that regulate it to ensure proper number, timing, and location of crossovers. Important questions remain however, particularly about how the cell orchestrates the multiple enzymatic activities that are each capable of acting on recombination intermediates. It is known that MutLgamma is the major, if not exclusive, activity responsible for resolving crossover-designated double Holliday junction intermediates, but Mus81-Mms4, STR (Sgs1-Top3-Rmi1), and Yen1 are all also able to take apart intermediates but with different net outcomes that often do not yield crossovers. A key barrier to progress has been the lack of a good experimental system to track the fates of recombination intermediates cleanly, because of confounding effects from ongoing new recombination initiation events that obscure what is happening at any one time.

This paper describes a clever and elegant experimental system that overcomes this barrier. The authors use established methods (ndt80 mutant) to arrest cells in pachytene and then to induce Cdc5 polo kinase to trigger events associated with pachytene exit, but they add in an inducible downregulation of DSB formation via sequestration of Rec104 protein. The authors' system prevents the confounding flux of new DSBs, allowing rigorous tracking of the fates of recombination intermediates. Using this system, the authors dissect the roles of the competing recombination intermediate-metabolizing activities as well as the role of the intermediate-stabilizing ZMM proteins.

These are technically demanding experiments but have been executed extremely well. The high quality data lend confidence in interpreting the results. The paper is clearly written. I had no suggestions for changes

Minor points:
Most figures show time as hours in SPM, but Fig. 1h says "in SPO" instead

Line 465: "joint molecule" or "JM" is probably missing between "high-MW" and "accumulation"

Version 1:

Reviewer comments:

Reviewer #1

(Remarks to the Author)
The authors have adressed my suggestions in the revised manuscript.
I have no further comments after this revised version. I think this is a beautiful piece of work and I can only praise the good job done by the authors.

Reviewer #2

(Remarks to the Author)
The authors have carefully addressed all of my concerns-it's a beautiful story.

Open Access This Peer Review File is licensed under a Creative Commons Attribution 4.0 International License, which permits use, sharing, adaptation, distribution and reproduction in any medium or format, as long as you give appropriate credit to the original author(s) and the source, provide a link to the Creative Commons license, and indicate if changes were made.
In cases where reviewers are anonymous, credit should be given to 'Anonymous Referee' and the source.
The images or other third party material in this Peer Review File are included in the article’s Creative Commons license, unless indicated otherwise in a credit line to the material. If material is not included in the article’s Creative Commons license and your intended use is not permitted by statutory regulation or exceeds the permitted use, you will need to obtain permission directly from the copyright holder.
To view a copy of this license, visit https://creativecommons.org/licenses/by/4.0/

We thank the reviewers for their encouraging and constructive feedback, which has helped us to improve the manuscript. We provide a point-by-point response to their comments below.

Note 1: *Author responses are highlighted in blue.*
Note 2: *Line numbers refer to the revised manuscript unless otherwise specified.*
Note 3: *Figure numbers refer to the revised manuscript unless otherwise specified.*

Reviewers' comments:

Reviewer #1:

This study addresses the regulation and resolution of meiotic recombination intermediates by combining an ndt80Δ arrest with rapamycin inducible anchor away depletion of Rec104. By temporally and conditionally controlling DSB formation, the authors achieve an unusually tight experimental window to interrogate the fate of ZMM-designated double Holliday junctions (dHJs) that give rise to a subset of crossover biased recombination events. From the outset, the sheer volume of data is striking and conveys the enormous experimental investment underlying the work, with time consuming, meticulously implemented assays and cleverly engineered genetic tools deployed throughout.

The work is distinguished by the elegant use of the anchor away system to acutely inactivate Rec104, providing a level of regulatory precision that represents the paper's key conceptual and technical advance. A comprehensive and rigorously controlled experimental framework—centered on one and two dimensional agarose gel analyses by southern blotting monitoring break formation, repair progression, and resolution outcomes—is of exceptional technical quality. Overall, the study is meticulously executed, conceptually coherent, and experimentally beautiful, leaving little to critique in terms of design or controls, and stands as an exemplary contribution to the field of meiotic recombination.

I have no major concerns. The following comments are suggestions to improve clarity and discussion.

We thank the reviewer for the positive comments and the acknowledgement of our efforts. We have revised the text to incorporate the suggested changes.

1- For readers familiar with two dimensional agarose gel analyses, the beauty of the gels stands out as an extremely well crafted experiment. I will nonetheless ask for a little more detail on how the quantification of the different Holliday junction species was performed on the two dimensional gels. The authors appropriately refer to reference 62 for the quantification procedure. However, to facilitate reading of the Results—for example Figure 1 (panels g and h)—and to better understand where the percentage of dHJs comes from, it would be useful to clarify what is defined as total DNA signal (all species and smearing, or only defined species). This could be visually indicated in Expanded Figure 1, where the complete gel with P1 and P2 spots is shown, for example by adding a simple schematic and a clarification in the figure legend.

This comment is not meant to question the validity of the quantifications, but reflects a genuine interest in making the analysis easier to follow directly from the paper, without the need to go deeper into referenced methods.

We thank the reviewer for the comments and the suggestion. We have included a panel in Supplementary Fig. 1e to illustrate the method of quantification, clarifying in the figure legend that the total DNA refers to the defined species. We have also expanded the Methods section (lines 672-677) to include the description of two-dimensional gel quantification.

2- This reviewer shares with the authors the view that one of the most original insights of this work is the identification of an Sgs1-independent function of Top3-Rmi1 in preserving the architecture of crossover designated intermediates. In the Discussion, the authors link this observation to changes in the accumulation of SUMOylated substrates. I would suggest expanding this line of discussion by considering some known properties of SUMO ligases in substrate recognition during DNA repair. Through their SAP domains, ligases such as Siz2 can scan dsDNA in search of substrates, and as described for RPA sumoylation, this can orient local SUMOylation at ssDNA–dsDNA boundaries. Top3, by altering the transitions of joint molecule intermediates, may influence the formation of "landing paths" for SUMO ligases or the availability of substrates at the boundaries of these remodeled species with increased ssDNA.

I think that identifying the positions of SUMO ligases, rather than measuring only global SUMOylation, could shed light on the mechanisms underlying the Top3 depletion phenotypes, and represents an interesting direction for future investigation.

We thank the reviewer for the great suggestion for future research, which we had not considered before. Indeed, changes in SUMOylation/distribution of SUMOylated substrates could be due to the recruitment of SUMO ligases to the remodelled species. We have revised the Discussion (lines 506-516) to include specific hypotheses explaining the Top3 depletion-induced changes to the SUMO landscape.

Reviewer #2:

This is an elegantly executed study that takes advantage of a very sophisticated system developed by the Matos lab to monitor pre-existing double-Holliday junction (dHJ) structures in yeast meiosis. A key feature of this system is that it removes potential complications associated with the continued initiation of recombination events in a mutant background (ndt80) that stabilizes dHJ structures in pachytene. The current work takes advantage of clever methods to prevent the induction of new recombination events in pachytene arrested cells and uses a construct to initiate dHJ resolution through induction of Cdc5 kinase. It also uses constructs to selectively degrade components of Sgs1-Top3-Rmi1, which were shown previously to act in multiple steps in meiotic recombination, including the prevention of aberrant DNA joint molecules and the promoting the formation of crossover-designated recombination intermediates. The goal of this work is to answer two questions posed by the authors in the abstract: 1. How are dHJs formed in the crossover-specific pathway directed toward resolution by the MutLgamma resolvase? 2. Is there a previously unknown role for the Sgs1-Top3-Rmi1 complex in maintaining the architecture of the dHJ in the crossover specific pathway?

The extremely detailed and sophisticated experiments presented in the manuscript are consistent with the authors conclusions. I agree with the authors that it supports “a hierarchy in dHJ processing, one that favors MutLgamma-directed crossovers while preserving alternative resolution routes to safeguard chromosome segregation.” The work also resolves a curiosity in the field in terms of whether ectopic expression of an activated Yen1 resolvase can lead to biased resolution of dHJs to form crossovers; the new work indicates that this is not the case. Lastly, it provides new information on an independent role for Top3-Rmi1 in preventing the conversion of dHJs in the crossover pathway from being processed into aberrant intermediates that cannot be acted upon by the MutLgamma resolvase. The comments listed below are aimed at improving the clarity of the manuscript.

We would like to thank the reviewer for the thorough feedback and suggestions. We have addressed the comments below.

1. The idea of a hierarchy in dHJ processing has been surmised for some time, beginning with genetic studies (some examples include de los Santos et al., Genetics 164:81; Argueso et al., Genetics 168:1805). I think that it would be appropriate to recognize these earlier studies. This doesn’t take away from how the current studies provide mechanistic evidence for a hierarchy argument. With this said I could see the value in speculating how the submitted work could be relevant to understanding meiotic recombination in higher eukaryotes. In baker’s yeast the MLH3 knockout only reduces crossing over by about 50% whereas in mouse MSH4/5 and MLH1/3 knockouts cause much more severe defects in crossing over. Also, in yeast the number of MutSgamma foci in pachytene is roughly equivalent to the ultimate number of meiotic crossovers; in mouse the number of Msh4-Msh5 foci is paired down significantly to approach the number of MutLgamma foci.

We thank the reviewer for these thoughtful suggestions. We have now incorporated references to earlier genetic studies (line 440) that helped shape the current view in the field that multiple pathways can access and process recombination intermediates. Our work builds on this foundation by providing direct mechanistic evidence supporting a hierarchy in dHJ processing. In addition, we have expanded the Discussion (lines 529–534) to consider how our findings may be relevant to meiotic recombination in higher eukaryotes.

2. The anchor away system performed in the ntd80null background is extremely powerful-it would be worth briefly stating how the kinetics of this system (e.g. the half-lives of DSBs, JMs, dHJs in the +rapamycin, + estradiol protocol presented in Fig. 1A) compares to the published half-lives in a standard wild-type SK1 time course that examines intermediates at the HIS4LEU2 hotspot.

We have amended the Results section (lines 144-149)

3. Figure 3F. A control for active Top3 (+rapamycin, -5-phenol-IAA, +beta-estradiol) is not included. I think that it’s important to include such a control within the same experiment to support the argument that the “NCOs that still arise in the mlh3null mus81null background depend on Top3….”

We agree with the reviewer that inclusion of this control is important. While the relevant samples (+rapamycin, −5-phenol-IAA, +β-estradiol) had been collected as part of the original experiment, they were not initially processed. We have now analyzed these samples and replaced the blots and quantifications in Fig. 3 accordingly. The results show that AID-tagged Top3 retains the ability to support joint molecule processing into noncrossovers in the absence of nucleases, thereby strengthening our conclusion that the residual NCO formation in the *mlh3Δ mus81Δ* background depends on Top3 activity.

4. Line 98. The work of Sanchez et al. is cited in the manuscript but it's worth adding a bit of context. Sanchez identified a non-canonical physical interaction between Exo1 and MutLgamma that is critical for MutLgamma activation, though the target of Cdc5 phosphorylation is unclear-it does not appear to be either Mlh1 or Mlh3.

We thank the reviewer for this helpful suggestion. We have revised the Introduction (lines 96–98) to provide additional context regarding the work of Sanchez et al., including the identification of a non-canonical interaction between Exo1 and MutLγ that is important for MutLγ activation. We also clarify that, although Cdc5 activity is required for this process, the relevant phosphorylation target remains unclear and does not appear to be Mlh1 or Mlh3.

5. Line 132 The FACS analysis only indicates that DNA premeiotic replication has been completed; it doesn't provide a direct measure that cells had reached pachytene at the time when Rec104 is anchored away. This sentence should be clarified. Can the authors provide more direct evidence that the various strains used in this study are in pachytene at T=7 hrs?

We thank the reviewer for this important point and agree that FACS analysis alone does not directly demonstrate pachytene entry. Due to the complexity of the experimental setup, where multiple inductions and depletions are performed in different genetic backgrounds, it was not feasible to collect and process chromosome spreads in parallel with all conditions analyzed in the manuscript. However, we performed meiotic time-course experiments of comparable scale using representative strains, including the most complex genotypes, to assess meiotic progression more directly. In these experiments, chromosome spreads collected at 6 h and 7 h in SPM were stained for Zip1 to monitor synapsis. Across all strains analyzed, the majority of cells exhibited fully synapsed chromosomes by 7 h.

We note that, in all experiments, we systematically monitor meiotic DNA replication kinetics by FACS as a key quality control. In our experience, replication kinetics provide a robust proxy for culture synchrony and for staging relative to meiotic induction. When replication profiles meet our stringent criteria, subsequent meiotic progression - including entry into pachytene - consistently follows with similar timing.

We have now included the Zip1-based analyses in Supplementary Fig. 1a–c and Supplementary Fig. 3c and revised the Results text accordingly (lines 129–136, 138-141, 249–252).

6. Line 160. Figure 1n. I think that the authors should spend a bit more effort defining the CO/NCO calculation. The calculation is a critical aspect of the paper that provides, at least for me, new evidence, through Rec104 anchor-away and Cdc5 activation experiments, that MutLgamma acts in a biased CO resolution pathway. The fact that this calculation involves measuring changes in CO and NCO levels after CDC5 activation is something that may not be obvious to many readers. This method essentially removes the ~80% of NCOs and the ~20% of COs that appear before CDC5 activation.

We agree with the reviewer and we have included a clearer explanation of the CO:NCO ratio (lines 173-177).

As an aside, it's interesting that NCOs rise at a faster rate when Rec104 is not anchored away and that this results in a CO/NCO ratio of ~1. The authors don't really comment on the continuous rise of NCOs-could this reflect a homeostasis mechanism where COs have already been designated and the increasing DSBs are shunted primarily to a NCO pathway?

We thank the reviewer for this interesting suggestion. We note that, in the absence of Cdc5 induction, both NCOs and dHJ-JMs increase with broadly similar kinetics, although the rise in

NCOs appears modestly steeper (Fig. 1j vs f). This could be interpreted as a potential indication of CO homeostasis. However, the difference is relatively subtle and, in our view, not sufficiently pronounced to warrant emphasis in the manuscript.
An important contributing factor to the continuous rise in NCOs is likely the progressive accumulation of cells that have generated a DSB at the *HIS4-LEU2* locus over time, leading to continued accumulation of both NCOs and JMs at the population level.

7. Beginning on line 475. The text positions Top3 as a “key architectural safeguard” but does not discuss how other Top3 interactors (e.g. Mer3, Altmannova et al., NAR 51:4363) may impact the protection of CO-designated intermediates. While work in this study suggests that Sgs1 does not play the safeguard role proposed for Top3, it’s worth noting that Chang et al. (EMBO J. 24:2024) reported that Sgs1 levels decrease in the absence of Top3, so other proteins could be impacted by the loss of Top3 function. There could be value of toning down the arguments presented in the Discussion to be open to a more complex mechanism.

We agree with the reviewer and have amended the Discussion (lines 500-504, 522).

Reviewer #3:

Crossing over between homologous chromosomes is needed in most species to promote accurate chromosome segregation at the first meiotic division. The field has learned much in the past couple of decades about the mechanisms that carry out crossover formation and that regulate it to ensure proper number, timing, and location of crossovers. Important questions remain however, particularly about how the cell orchestrates the multiple enzymatic activities that are each capable of acting on recombination intermediates. It is known that MutLgamma is the major, if not exclusive, activity responsible for resolving crossover-designated double Holliday junction intermediates, but Mus81-Mms4, STR (Sgs1-Top3-Rmi1), and Yen1 are all also able to take apart intermediates but with different net outcomes that often do not yield crossovers. A key barrier to progress has been the lack of a good experimental system to track the fates of recombination intermediates cleanly, because of confounding effects from ongoing new recombination initiation events that obscure what is happening at any one time.

This paper describes a clever and elegant experimental system that overcomes this barrier. The authors use established methods (ndt80 mutant) to arrest cells in pachytene and then to induce Cdc5 polo kinase to trigger events associated with pachytene exit, but they add in an inducible downregulation of DSB formation via sequestration of Rec104 protein. The authors’ system prevents the confounding flux of new DSBs, allowing rigorous tracking of the fates of recombination intermediates. Using this system, the authors dissect the roles of the competing recombination intermediate-metabolizing activities as well as the role of the intermediate-stabilizing ZMM proteins.

These are technically demanding experiments but have been executed extremely well. The high quality data lend confidence in interpreting the results. The paper is clearly written. I had no suggestions for changes

We thank the reviewer for the positive comments and the appreciation of our efforts!

Minor points:
Most figures show time as hours in SPM, but Fig. 1h says “in SPO” instead

Line 465: “joint molecule” or “JM” is probably missing between “high-MW” and “accumulation”

We thank the reviewer for pointing out the typos, they have been amended.

We thank the reviewers for their overall feedback and comments, which has helped us solidify our findings and polish the manuscript.

Note 1: *Author responses are highlighted in blue.*

Reviewer's comments:

Reviewer #1

The authors have adressed my suggestions in the revised manuscript.
I have no further comments after this revised version. I think this is a beautiful piece of work and I can only praise the good job done by the authors.

We are grateful to the reviewer for their positive remarks and the appreciation of our work

Reviewer #2 (Remarks to the Author):

The authors have carefully addressed all of my concerns-it's a beautiful story.

We thank the reviewer for their comments and recognition of our work.
